# Senescent Cell‐Derived Extracellular Vesicles in Therapy‐Induced Senescence: Immune Remodelling and Emerging Links to Mitochondrial Stress in Cancer

**DOI:** 10.1002/jex2.70193

**Published:** 2026-10-15

**Authors:** Yongrui Qian, Zirui Weng, Na Li, Yun Zhou, Hailong Pei

**Affiliations:** ^1^ State Key Laboratory of Radiation Medicine and Protection, School of Radiation Medicine and Protection Suzhou Medical College of Soochow University Suzhou Jiangsu P.R. China; ^2^ Medical Centre for Digestive Diseases The Second Affiliated Hospital of Nanjing Medical University Nanjing China; ^3^ Department of Radiation Oncology Xuzhou Central Hospital, Southeast University Xuzhou China

**Keywords:** cancer recurrence, cGAS–STING, extracellular vesicles, mitochondrial DNA, senescent cell‐derived extracellular vesicles, therapy‐induced senescence, tumour immunity

## Abstract

Cancer therapy can induce cellular senescence and remodel the tumour microenvironment through the senescence‐associated secretory phenotype (SASP), including its membrane‐enclosed component, senescent cell‐derived extracellular vesicles (senEVs). This review summarizes senEV biogenesis, cargo‐dependent functions, and immune‐remodelling effects while distinguishing mechanisms directly demonstrated in experimentally validated senescent‐cell EV models from contextual evidence derived from broader EV and mitochondrial‐stress studies. Direct evidence shows that senEVs can support immune‐mediated clearance in some models or promote tumour growth, chemoresistance, and polymorphonuclear myeloid‐derived suppressor cell (PMN‐MDSC)‐mediated immunosuppression in others. We further evaluate mitochondrial stress and EV‐associated mitochondrial DNA as an emerging mechanistic framework that may contribute to these divergent outcomes, rather than as an established general regulator of senEV activity. Finally, we discuss candidate intervention strategies, evidence gaps, and the experimental requirements needed to define how donor‐cell state, cargo composition, recipient‐cell identity, treatment context and time after treatment may shape senEV function.

## 1 Introduction: The ‘Senescence‐Associated Secretory System’ Under Therapeutic Stress and Particulate SASP

Cancer therapies, including radiotherapy, chemotherapy, targeted therapy and immunotherapy, not only eliminate tumour cells but also inevitably impose persistent cellular stress, such as DNA damage, oxidative stress (Dong et al. 2024; Song et al. 2023; Ding et al. 2025), metabolic pressure (Dong et al. 2024; Ding et al. 2025; Wang et al. 2024) and disruption of organelle homeostasis (Tanaka and Takahashi 2021; Carles‐Fontana et al. 2022; Gade et al. 2025). These stresses can drive tumour or stromal cells into a senescent state characterized by irreversible growth arrest (Tanaka and Takahashi 2021; Okawa et al. 2024; Misawa et al. 2020; Urbanelli et al. 2016). Depending on the cellular and temporal context, senescent cells can either enhance immune surveillance, tissue repair and immune‐mediated clearance (Wang et al. 2024; Ziglari et al. 2025; Almeida and Melo 2025; Kang et al. 2011; Xue et al. 2007), or sustain chronic inflammation, myeloid immunosuppression and immune evasion, thereby creating a niche for residual‐cell regrowth, recurrence and treatment resistance (Dong et al. 2024; Song et al. 2023; Ding et al. 2025; Liu et al. 2024; Lai et al. 2025; Zhang et al. 2024).

Senescent cells develop a senescence‐associated secretory phenotype (SASP) that classically comprises soluble cytokines, chemokines, growth factors and proteases, including matrix metalloproteinases (Wang et al. 2024; van Deursen 2014; Kuilman et al. 2008; Rodier et al. 2009). In this review, these freely secreted mediators are referred to as the soluble SASP, whereas extracellular vesicles (EVs) released by senescent cells are considered a membrane‐enclosed, particulate component of senescence‐associated intercellular communication (Wang et al. 2024; Tanaka and Takahashi 2021; Okawa et al. 2024; Misawa et al. 2020; Urbanelli et al. 2016). These two modes of signalling are distinct but not mutually exclusive, because molecules commonly classified as soluble SASP factors can also be incorporated into the EV lumen or associated with the EV surface and thereby delivered to recipient cells (Fitzgerald et al. 2018). EVs provide membrane‐enclosed, structured packaging that can protect their cargo in biofluids and facilitate long‐distance delivery and selective uptake by recipient cells (Welsh et al. 2024; Thery et al. 2018; van Niel et al. 2018; Colombo et al. 2014; Kalluri and LeBleu 2020). EVs can package and transport proteins, lipids, metabolites and damage‐associated molecular patterns (DAMPs), including nuclear DNA and mitochondrial DNA (mtDNA) (Welsh et al. 2024; Thery et al. 2018; van Niel et al. 2018; Colombo et al. 2014; Kalluri and LeBleu 2020; Kalluri and McAndrews 2023), thereby extending the influence of senescence‐like cells from local paracrine signalling to transmissible systemic regulation (Tanaka and Takahashi 2021; Okawa et al. 2024; Misawa et al. 2020; Urbanelli et al. 2016). Importantly, the capacity to promote tumour suppression or immune‐mediated clearance is not unique to senEVs. Depending on its composition and temporal context, the soluble SASP can reinforce senescence and recruit immune cells that eliminate senescent or premalignant cells (Kang et al. 2011; Xue et al. 2007; Kuilman et al. 2008; Rodier et al. 2009). Direct evidence shows that senEVs can promote immune‐mediated clearance of senescent cells (Ziglari et al. 2025) or, in other models, enhance recipient tumour‐cell growth and immunosuppression mediated by polymorphonuclear myeloid‐derived suppressor cells (PMN‐MDSCs) (Lai et al. 2025; Zhang et al. 2024; Takasugi et al. 2017). Broader studies of tumour‐derived EVs support additional roles in immune tolerance and treatment resistance, but these findings should not be attributed specifically to senEVs unless the donor‐cell senescent state has been validated (Kalluri and McAndrews 2023; Ahn et al. 2025; Kuang et al. 2025; Spada and Ganguly 2025; Yue et al. 2023). Thus, senEVs and soluble SASP factors share context‐dependent functional duality, whereas senEVs differ primarily in their membrane‐enclosed cargo packaging and delivery, which can influence cargo stability, biodistribution and uptake by recipient cells (Welsh et al. 2024; Thery et al. 2018; van Niel et al. 2018; Colombo et al. 2014; Kalluri and LeBleu 2020).

To avoid ambiguity, we use EVs as the umbrella term for naturally released, lipid‐bilayer‐delimited particles that cannot replicate (Welsh et al. 2024; Thery et al. 2018). When their biogenetic origin has not been directly demonstrated, we use operational size‐based terms, principally small EVs (sEVs), rather than assigning a specific biogenetic subtype. Exosomes refer specifically to endosome‐derived EVs released following the fusion of multivesicular bodies (MVBs) with the plasma membrane, whereas ectosomes, also commonly termed microvesicles, bud directly from the plasma membrane (van Niel et al. 2018; Colombo et al. 2014). The term exosome‐like sEVs is used only for small EVs with features commonly associated with exosomes but without definitive evidence of endosomal origin. In this review, senescent cell‐derived EVs (senEVs) denotes EVs released by experimentally validated senescent cells, regardless of their biogenetic subtype. Throughout this review, the term mitochondrial cargo‐containing extracellular vesicles (mitoEVs) is used solely as an operational shorthand for EVs in which mitochondrial components, including mitochondrial DNA, RNA, proteins, lipids, mitochondrial fragments, or, in some reports, intact mitochondria, have been detected. The term describes cargo composition and does not by itself establish that the vesicle membrane is derived directly from mitochondria. By contrast, mitochondria‐derived vesicles (MDVs) refers specifically to intracellular vesicles that bud from mitochondria and should not be used interchangeably with extracellular mitoEVs. Study‐specific terms such as ‘mitochondria‐encapsulating EVs’ or ‘EV‐Mito’ are retained only when describing the corresponding source studies (Iorio et al. 2024; Sheng et al. 2025).

This leads to the central question of this review: why do senescent cell‐derived EVs (senEVs) show opposite effects under different treatment modalities, time windows and immune niches? Which candidate factors may contribute to this functional directionality? We examine the hypothesis that mitochondrial stress and the leakage of related nucleic acids, particularly mtDNA, together with DNA‐sensing pathways, may alter EV cargo loading and the innate immune threshold of recipient cells, thereby biasing outcomes toward either clearance or escape (Carles‐Fontana et al. 2022; Gade et al. 2025; Lai et al. 2025; Iorio et al. 2024; Sheng et al. 2025; Dou et al. 2017; Gluck et al. 2017; Li et al. 2024). This review focuses on how therapy‐induced senescence (TIS) reshapes EV‐mediated signalling, with particular attention to mitochondrial stress, immune remodelling and translational implications (Song et al. 2023; Ding et al. 2025; Ziglari et al. 2025; Almeida and Melo 2025; Kang et al. 2011; Xue et al. 2007; Liu et al. 2024; Lai et al. 2025; Zhang et al. 2024; Takasugi et al. 2017; Saleh 2024; Wang et al. 2025; Xiao et al. 2023). It further synthesizes evidence linking these observations to mechanisms of immune regulation and mitochondrial signalling (Carles‐Fontana et al. 2022; Gade et al. 2025; Kang et al. 2011; Xue et al. 2007; Kalluri and McAndrews 2023; Ahn et al. 2025; Kuang et al. 2025; Spada and Ganguly 2025; Yue et al. 2023; Iorio et al. 2024; Sheng et al. 2025; Dou et al. 2017; Gluck et al. 2017; Li et al. 2024; Alfonzo et al. 2022; Takasugi 2018; Wolfers et al. 2001; Obregon et al. 2006; Van Niel et al. 2003; Segura et al. 2007; Nolte‐’t Hoen et al. 2009; Sheldon et al. 2010), and discusses potentially translatable interventions and candidate senEV‐associated biomarkers (Carles‐Fontana et al. 2022; Gade et al. 2025; Iorio et al. 2024; Sheng et al. 2025; Li et al. 2024; Choezom and Gross 2022; Hitomi et al. 2020; Menck et al. 2017; Pacifico et al. 2024; Simpson et al. 2009; Meng et al. 2023; Lee et al. 2022) (Figure 1).

**FIGURE 1:**
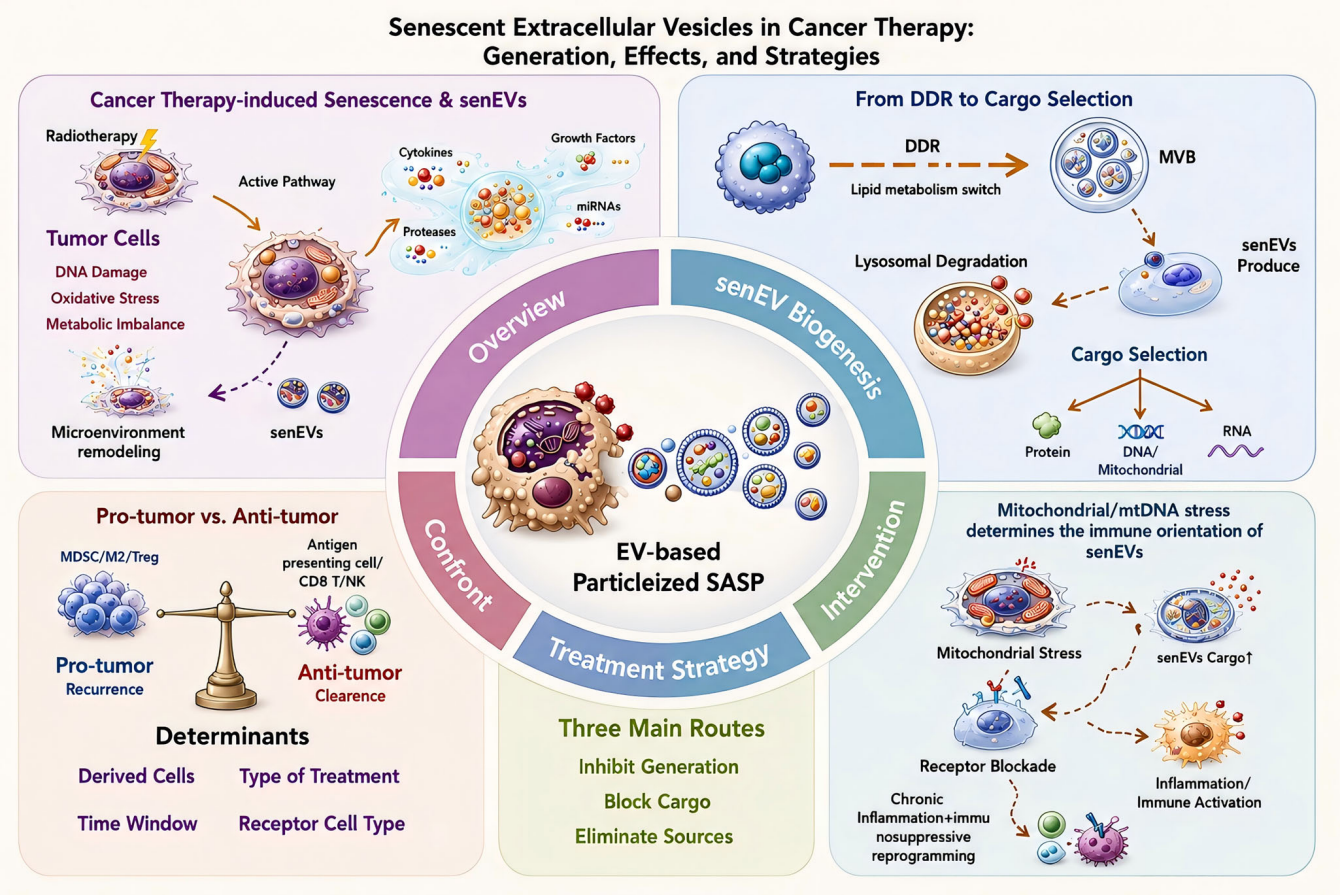
Overview of therapy‐induced senescence and senEV‐mediated immune remodelling in the tumour microenvironment. Cancer therapies such as chemotherapy, radiotherapy, targeted therapy, and immunotherapy can induce persistent cellular stress and therapy‐induced senescence in tumour cells and stromal cells within the tumour microenvironment. Senescent cells subsequently develop a complex senescence‐associated secretory phenotype (SASP), including the release of soluble inflammatory mediators and senescent cell‐derived extracellular vesicles (senEVs). Compared with soluble SASP factors, senEVs provide a more stable and spatially transferable signalling platform capable of delivering proteins, nucleic acids, lipids, and mitochondrial components to surrounding recipient cells. Following uptake by immune and stromal cells, senEV‐associated signals may influence dendritic‐cell activation, macrophage polarization, T‐cell responses, and myeloid‐cell remodelling in a context‐dependent manner. These dynamic interactions may either support transient antitumour immune surveillance and residual tumour‐cell clearance or contribute to chronic immunosuppressive remodelling, immune escape and recurrence following therapy. The figure summarizes the overall framework linking therapy‐induced senescence, senEV signalling, and divergent immune outcomes discussed throughout this review.

## 2 Definitions and Evidence Framework

### 2.1 Definitions of Cellular Senescence, TIS, EVs and senEVs

Cellular senescence generally refers to a state of irreversible growth arrest that cells enter in response to persistent stress. Its core feature is not merely a ‘temporary cessation of division,’ but rather a state characterized by stable cell‐cycle exit, sustained activation of stress pathways, and reprogramming of the secretory system (van Deursen 2014; Kuilman et al. 2008; Rodier et al. 2009; Baker et al. 2011). Unlike quiescence, which is a reversible pause in proliferation, senescence is characterized by irreversible growth arrest and extensive phenotypic remodelling. While remaining viable, senescent cells undergo systematic changes in morphology, metabolism, chromatin state and secretory activity. Many of their context‐dependent effects are mediated by the SASP, which includes soluble cytokines, chemokines, growth factors and proteases, as well as EV‐associated cargo (Wang et al. 2024; van Deursen 2014; Kuilman et al. 2008; Rodier et al. 2009). EVs released by experimentally validated senescent cells are termed senEVs in this review and are discussed as a membrane‐enclosed, or ‘particulate,’ component of the SASP. It should be emphasized that senescence is not a single phenotype: different triggers (replicative senescence, oncogene‐induced senescence, oxidative stress, radiotherapy/chemotherapy, etc.) and different cell types can generate marked heterogeneity. Therefore, in EV research, ‘senescence’ must first be a clearly validated and traceable experimental state, rather than an inference based on a single marker (Wang et al. 2024; van Deursen 2014; Kuilman et al. 2008; Rodier et al. 2009; Baker et al. 2011).

TIS is the most common senescence‐like program in the context of cancer therapy, and also the one that requires the strictest definition (Dong et al. 2024; Song et al. 2023; Ding et al. 2025; Liu et al. 2024; Lai et al. 2025; Zhang et al. 2024; Saleh 2024; Wang et al. 2025; Xiao et al. 2023). DNA damage, replication stress, and metabolic/oxidative stress caused by radiotherapy, chemotherapy, targeted therapy, and other treatments can drive tumour cells or stromal cells into TIS. The key significance of TIS is that it often occurs in cell populations that survive treatment. TIS and senEV release can facilitate immune‐mediated clearance in some models (Ziglari et al. 2025; Kang et al. 2011; Xue et al. 2007), whereas persistent senescent cells or their EVs can promote tumour progression and immunosuppression in others (Lai et al. 2025; Zhang et al. 2024; Takasugi et al. 2017). These outcomes vary with the senescence inducer, cell type, and sampling time and should not be interpreted as a universal acute‐versus‐chronic sequence. Therefore, when discussing ‘senEVs’ in this review, we assume by default that their sources may include not only senescent cells in the traditional sense, but also TIS cell populations that have entered senescence‐like reprogramming under therapeutic stress.

As defined in the Introduction, EV is used here as an umbrella term, whereas subtype‐specific terminology is applied only when supported by evidence of particle size or biogenesis (Welsh et al. 2024; Thery et al. 2018; van Niel et al. 2018; Colombo et al. 2014; Kalluri and LeBleu 2020). A consensus in the EV field is that, under most experimental conditions, it is difficult to ‘prove their biogenetic origin’ solely on the basis of isolated products (for example, to strictly distinguish exosomes from microvesicles). Therefore, the International Society for Extracellular Vesicles (ISEV) advocates more operational and reproducible modes of nomenclature and classification, and the MISEV guidelines specify the minimum information that should be reported in EV studies (Welsh et al. 2024; Thery et al. 2018; van Niel et al. 2018; Colombo et al. 2014; Kalluri and LeBleu 2020; Lotvall et al. 2014). Specifically, terms based on physical properties (mainly size range), such as small EV (sEV) and medium/large EV (m/lEV), are recommended unless sufficient evidence can be provided to demonstrate origin from a specific biogenetic pathway (Welsh et al. 2024; Thery et al. 2018; van Niel et al. 2018; Colombo et al. 2014; Kalluri and LeBleu 2020; Lotvall et al. 2014). In tumour samples, EVs can originate from both malignant and non‐malignant cells, and tumour‐cell‐derived EVs are not necessarily senEVs. In this review, the term senEVs is restricted to EVs whose cellular source has been experimentally validated as senescent; EVs released by tumour cells without such validation are considered part of the broader category of tumour‐derived EVs (Welsh et al. 2024; Thery et al. 2018; van Niel et al. 2018; Colombo et al. 2014; Kalluri and LeBleu 2020; Kalluri and McAndrews 2023).

### 2.2 Evidence Framework of this Review: From Basic Mechanisms to Translational Strategies

This review organizes the available literature from basic mechanisms to translational implications and distinguishes findings obtained directly from experimentally validated senescent‐cell EV models from contextual evidence derived from broader studies of cellular senescence, tumour‐derived EVs, or mitochondrial cargo‐containing EVs (Dong et al. 2024; Song et al. 2023; Ding et al. 2025; Wang et al. 2024; Liu et al. 2024; Lai et al. 2025; Zhang et al. 2024; Welsh et al. 2024; Thery et al. 2018; van Niel et al. 2018; Colombo et al. 2014; Kalluri and LeBleu 2020; Saleh 2024; Wang et al. 2025; Xiao et al. 2023; Lotvall et al. 2014; Raposo and Stoorvogel 2013). This distinction helps avoid treating evidence obtained from different experimental contexts as equivalent. For clarity, findings directly demonstrated using EVs from experimentally validated senescent cells are described as senEV‐specific evidence. Findings derived from broader EV, mitochondrial, or tumour‐immunity studies are treated as contextual evidence, whereas integrative interpretations proposed in this review are explicitly labelled as working hypotheses. These evidence categories are also encoded visually throughout the figures using explicit evidence labels and explanatory legends. In Figures 2 and 3, green ‘DIRECT’ labels identify senEV‐specific findings, whereas grey ‘CONTEXTUAL’ or ‘PROPOSED’ labels identify extrapolated or hypothetical relationships. In Figure 4, D/P labels indicate the evidence status of individual cargos, and recipient‐to‐outcome links are distinguished using solid or dashed arrows where appropriate. Figure 5 is explicitly presented as a working model, and its curves are illustrative rather than longitudinal experimental measurements. These visual conventions indicate the strength and context of evidence rather than the direction or magnitude of an effect.

1) Mechanistic core

**FIGURE 2:**
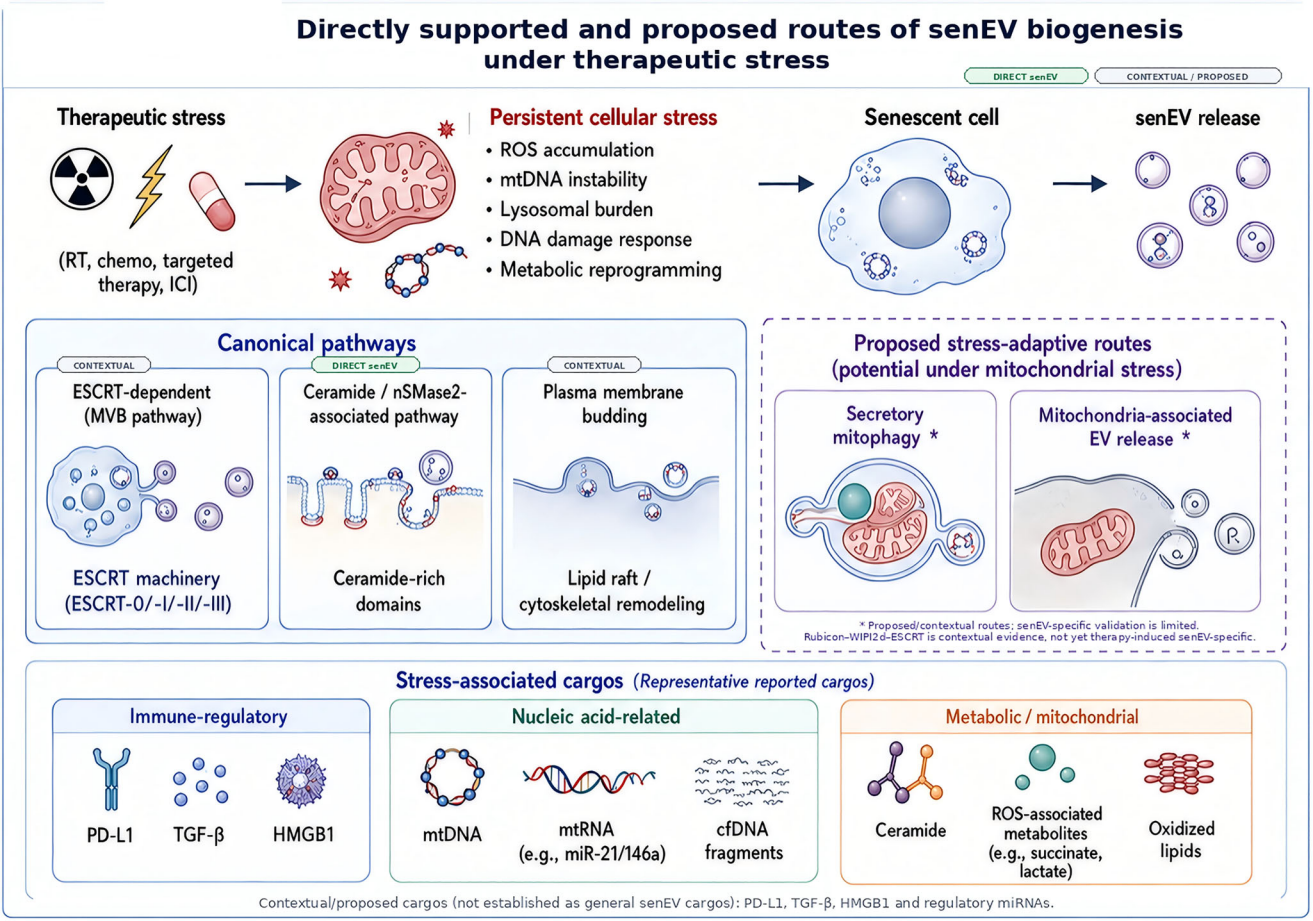
Directly supported and proposed routes of senEV biogenesis under therapeutic stress. The original schematic structure is retained, while evidence status is indicated using explicit labels. The ceramide/nSMase2‐associated pathway is marked as directly supported in senEV models, whereas general ESCRT‐dependent multivesicular‐body formation and plasma‐membrane budding are presented as contextual mechanisms. Secretory mitophagy, mitochondria‐associated EV release and the Rubicon–WIPI2d–ESCRT relationship are shown as proposed or contextual routes requiring senEV‐specific validation. PD‐L1, TGF‐β, HMGB1, and regulatory miRNAs are retained as representative contextual or proposed cargos rather than established general senEV cargos.

**FIGURE 3:**
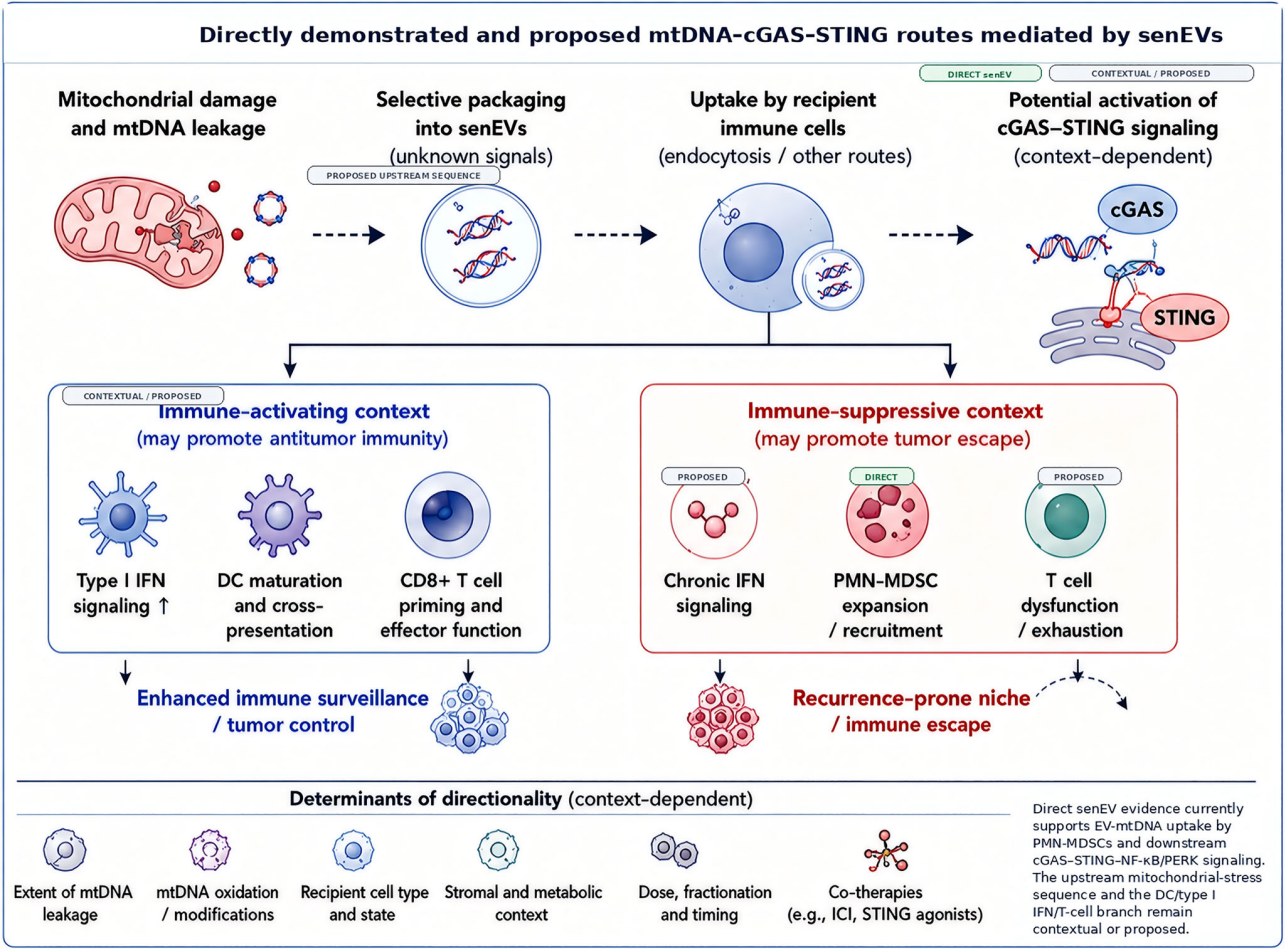
Directly demonstrated and proposed mtDNA–cGAS–STING routes mediated by senEVs. Evidence‐status labels distinguish experimentally supported findings from contextual or proposed relationships. Direct senEV‐specific evidence supports the transfer of EV‐associated mtDNA to PMN‐MDSCs and subsequent activation of cGAS–STING–NF‐κB/PERK signalling. By contrast, the upstream sequence linking mitochondrial stress to selective mtDNA loading, as well as the dendritic‐cell/type I interferon/T‐cell‐priming and T‐cell‐dysfunction branches, is presented as contextual or proposed. The figure therefore does not establish mitochondrial stress as a general upstream regulator of senEV‐mediated immune effects.

**FIGURE 4:**
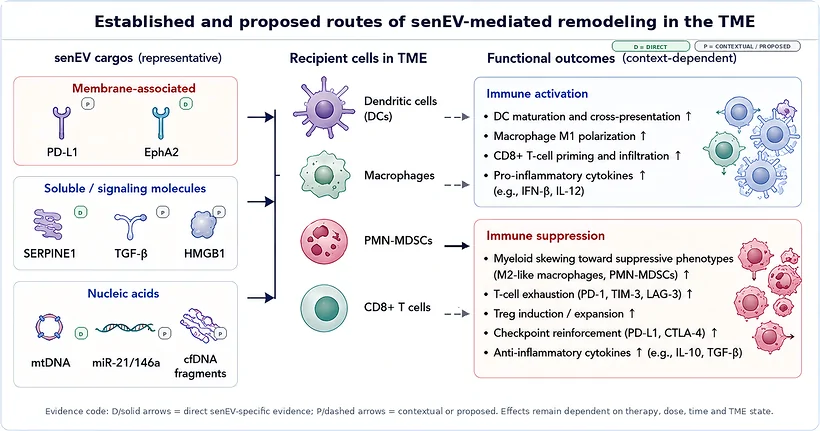
Established and proposed routes of senEV‐mediated remodelling in the tumour microenvironment. D labels identify cargos with direct senEV‐specific support in the cited experimental models, including EphA2, SERPINE1 and mtDNA, whereas P labels identify contextual or proposed cargos, including PD‐L1, TGF‐β, HMGB1, regulatory miRNAs and cfDNA fragments. Among the recipient‐to‐outcome links, the solid arrow indicates the directly supported mtDNA–PMN‐MDSC immunosuppressive pathway, whereas dashed arrows indicate contextual or proposed dendritic‐cell, macrophage and T‐cell responses. Dendritic‐cell cross‐presentation and T‐cell exhaustion have not been established as general senEV‐specific mechanisms. Evidence status is model‐specific and does not imply that the indicated cargos or effects are universal features of senEVs.

**FIGURE 5:**
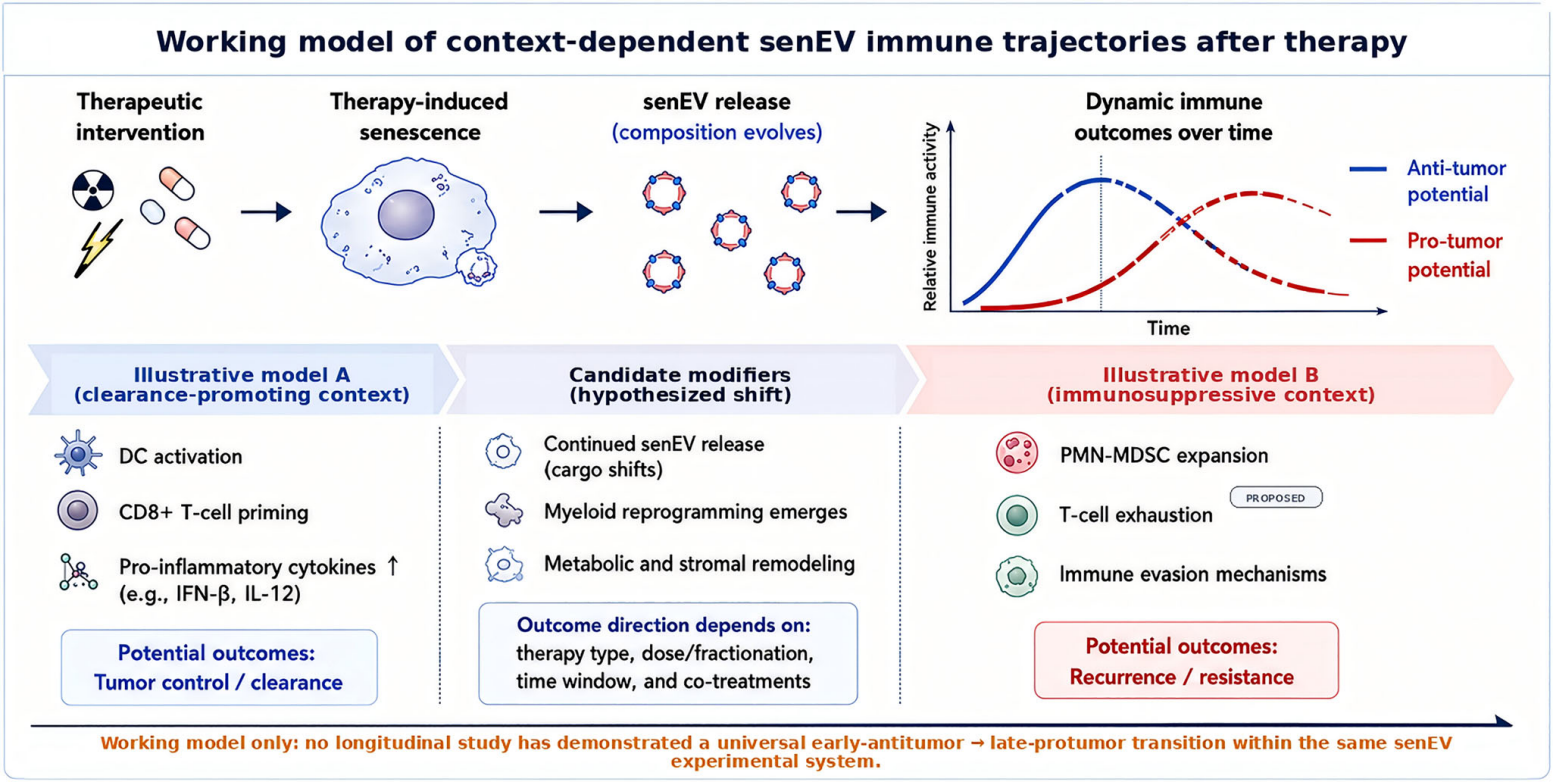
Working model of context‐dependent senEV immune trajectories after therapy. The original time‐axis schematic is retained for conceptual organization, but the blue and red curves are illustrative and do not represent quantitative or longitudinal experimental measurements. Clearance‐promoting senEV activity and mtDNA–PMN‐MDSC‐mediated immunosuppression have been demonstrated in separate experimental systems systems, whereas their placement along a shared time axis and the intervening shift remain hypothetical. The model does not imply that senEVs universally transition from antitumour to protumour activity over time; longitudinal validation within the same experimental system is required.

This review first considers why senescent cells increase EV release, how cargo may be selectively loaded, and which upstream pathways may contribute to EV biogenesis. The focus includes: (a) DNA damage response (DDR)‐related pathways and lipid metabolism, particularly direct senEV evidence linking DNA damage to p53‐associated EV release and to the ceramide/neutral sphingomyelinase 2 (nSMase2) axis (Choezom and Gross 2022; Hitomi et al. 2020; Menck et al. 2017; Pacifico et al. 2024); and (b) the innate nucleic acid‐sensing axis, including contextual evidence linking cyclic GMP–AMP synthase (cGAS)–stimulator of interferon genes (STING) signalling to abnormal cytosolic DNA or mtDNA (Dou et al. 2017; Gluck et al. 2017). These pathways provide candidate, rather than universally established, intervention nodes for the discussion that follows.

2) Contextual oncology evidence

The second level considers direct evidence from validated senescent tumour‐ or stromal‐cell models (Ziglari et al. 2025; Lai et al. 2025; Zhang et al. 2024; Takasugi et al. 2017), while broader studies of tumour‐derived EVs are used only as contextual evidence for potential effects on the residual‐tumour microenvironment, immune evasion, recurrence and responses to immunotherapy (Xue et al. 2007; Kalluri and McAndrews 2023; Ahn et al. 2025; Kuang et al. 2025; Spada and Ganguly 2025). Time after treatment and EV cellular source are candidate modifiers; however, no longitudinal study has demonstrated a reproducible transition from an acute clearance‐promoting senEV state to a chronic recurrence‐promoting state within the same experimental model.

3) The mitochondria–mtDNA–immunity axis

The third level evaluates mitochondrial stress as a candidate explanatory framework for divergent senEV outcomes. Direct senEV‐specific evidence currently supports EV‐associated mtDNA transfer to PMN‐MDSCs and downstream immunosuppression (Lai et al. 2025), whereas the broader proposition that mitochondrial stress governs senEV cargo selection and immune directionality across cell types and treatments remains a working hypothesis.

4) Translational strategies

The final level addresses ‘how to intervene,’ including inhibition of EV biogenesis/release (such as nSMase2‐related pathways), neutralization of key cargos, blockade of recipient uptake, and the potential and risks of combining these approaches with senotherapy or the ‘one‐two punch’ strategy (Choezom and Gross 2022; Hitomi et al. 2020; Menck et al. 2017; Pacifico et al. 2024). This part places greater emphasis on the strategic logic of designing interventions around treatment windows, tissue specificity and controllable side effects (Choezom and Gross 2022; Hitomi et al. 2020; Menck et al. 2017; Pacifico et al. 2024).

To distinguish evidence generated directly in senescence models from mechanisms extrapolated from the broader EV field, representative original studies in which donor‐cell senescence was experimentally validated, or in which a senescence contribution to circulating EVs was directly tested, are summarized in Table 1. General EV studies are used only as contextual evidence in the mechanistic sections below.

**TABLE 1:** Experimental design and strength of evidence in representative senEV studies.

Study/senescence model and donor cells	Criteria used to validate cellular senescence	EV isolation and characterization	EV subtype	Cargo/feature	Cargo‐specific causality tested?	Reported biological effect/mechanism	In vivo validation of EV‐dependent effect?
Lehmann et al. (2008) [new Ref. A] Ionizing‐radiation‐induced premature senescence; 22Rv1 and LNCaP prostate cancer cells	SA‐β‐gal positivity; sustained p53/p21 response and growth arrest after irradiation; dominant‐negative p53 comparison	Clarification followed by 100,000 × *g* ultracentrifugation and PBS wash; ultrastructural/particle and exosomal‐protein analyses as reported	Exosome‐like EVs	No causal cargo identified; EV‐release phenotype linked to p53 competence	No—p53 dependence of EV release was tested, but no individual EV cargo was linked to a recipient‐cell phenotype	Radiation increased exosome‐like EV release; dominant‐negative p53 attenuated senescence and vesicle release	No
Takasugi et al. (2017) Replicative, Ras‐ or doxorubicin‐induced senescence; TIG‐3 and RPE‐1 cells	SA‐β‐gal; p21 and phospho‐p53; replicative/oncogene/drug‐induced arrest across multiple models	Differential centrifugation, 0.22‐µm filtration, 30% sucrose/D_2_O cushion and 100,000 × *g* washes; NTA; Alix/CD9 and other EV‐marker immunoblotting; proteomics	Exosome‐like sEVs	EphA2	Yes—EphA2 knockdown, rescue and overexpression; ephrin‐A1 blockade	EphA2–ephrin‐A1 reverse signalling promoted proliferation of selected cancer‐cell lines; ROS/PTP1B signalling increased EphA2 loading	No
Borghesan et al. (2019) [new Ref. B] Oncogene‐ and DNA‐damage‐induced senescence; human fibroblasts	Stable proliferative arrest/EdU loss; SA‐β‐gal; p16/p21 and DNA‐damage/SASP markers across senescence models	Serial ultracentrifugation with density‐gradient validation; TEM, NTA, EV‐marker immunoblotting (CD63, TSG101, ALIX) and proteomics	sEVs	IFITM3	Yes/partial—donor‐cell IFITM3 depletion attenuated and EV IFITM3 enrichment promoted paracrine senescence	sEVs transmitted a senescence‐associated transcriptional and growth‐arrest phenotype to recipient cells; IFITM3 was partially responsible	No functional in vivo cargo validation (in vivo CD63/MVB association was examined)
Hitomi et al. (2020) DNA‐damage‐associated senescence; TIG‐3 human fibroblasts	DNA‐damage markers (γH2AX and pST/Q), p21/SA‐β‐gal and senescence‐associated growth phenotype as reported	Differential centrifugation, filtration, sucrose‐cushion ultracentrifugation and washing; NTA and EV‐marker analyses	SA‐EVs/sEVs	Cytoplasmic DNA fragments	Partial—SMS2/nSMase2, EV‐biogenesis and autophagy perturbations linked DNA disposal to donor‐cell inflammation/apoptosis; no recipient‐cell cargo rescue	SMS2↓/nSMase2↑ activated ceramide‐dependent EV release, supporting cytoplasmic‐DNA disposal and limiting excessive donor‐cell inflammation/apoptosis	No
Miyazoe et al. (2020) Etoposide‐induced senescence; human hepatic stellate cells	Irreversible cell‐cycle arrest/EdU loss; 53BP1 and p21 upregulation; increased SA‐β‐gal	Ultracentrifugation; nanoparticle tracking analysis; EV quantity compared between control and senescent donors	EVs	Not identified	No	senEVs increased EGF secretion from differentiated THP‐1 macrophage‐like cells and increased hepatoma‐cell viability in co‐culture	No
Meng et al. (2023) Replicative, irradiation‐ or etoposide‐induced senescence; WI‐38 fibroblasts	Multiple senescence inducers; SA‐β‐gal/growth‐arrest and senescence‐marker validation as reported	Differential ultracentrifugation and EV purification; electron microscopy, particle sizing/quantification, EV‐marker analysis and surface proteomics	EVs	Surface DPP4	Yes—DPP4 reduction/inhibition increased uptake; DPP4 overexpression reduced uptake	EV‐surface DPP4 acted as an uptake repressor in proliferating fibroblasts and HeLa cells	No
Zhang et al. (2024) Irinotecan‐ or radiation‐induced senescence; HCT116 and RKO colorectal cancer cells	SA‐β‐gal; EdU and colony‐formation loss; G_2_/M arrest; p53, p21, γH2AX and SASP‐marker induction	Differential centrifugation, 0.22‐µm filtration and 100,000 × *g* ultracentrifugation/wash; TEM, NTA, EV‐marker immunoblotting with negative‐control marker; uptake assays	sEV‐enriched EVs	SERPINE1	Yes—pharmacological blockade, donor/EV manipulation, protection/transfer and downstream rescue experiments	EV‐associated SERPINE1 bound p65, promoted NF‐κB activation and enhanced colorectal‐cancer proliferation, migration, invasion and growth	Yes—xenograft and EV‐transfer/intervention experiments
Ziglari et al. (2025) Paclitaxel‐ or cisplatin‐induced senescent murine tumour cells; immunocompetent engraftment models	Persistent non‐proliferative state after drug withdrawal; SPiDER‐β‐gal; reduced BrdU incorporation; cell‐death controls	Differential ultracentrifugation with wash; TEM, NTA, flotillin‐1 immunoblotting and quantitative proteomics	senEVs	APC–T‐cell adhesion‐related surface ligands (candidate set; no single causal cargo)	No single cargo—EV dependence tested with Rab35‐DN suppression and EV add‐back	senEVs recruited MHC‐II‐positive APCs and supported CCR2‐positive CD4‐positive TH17‐mediated immune surveillance and recurrence suppression	Yes—immunocompetent engraftment, recurrence, EV‐inhibition and EV‐complementation experiments
Lai et al. (2025) Primary senescence and therapy‐induced senescence; prostate‐cancer models	Multiple senescence models with stable arrest/SA‐β‐gal and treatment‐appropriate senescence markers as reported	CD63‐based EV labelling/fractionation; EV‐associated mtDNA quantification and protection/localization analyses; EV uptake and pathway assays	mtDNA‐containing EVs	mtDNA	Yes—mtDNA/VDAC‐dependent release and recipient cGAS–STING pathway perturbations linked the cargo to PMN‐MDSC function	EV‐associated mtDNA activated cGAS–STING–NF‐κB/PERK signalling in PMN‐MDSCs, enhanced immunosuppression and promoted tumour progression	Yes—genetic/therapy prostate‐tumour models and pathway‐intervention experiments
Jia et al. (2026) [new Ref. D] 5‐FU‐ or oxaliplatin‐induced senescent HUVECs	SA‐β‐gal; pRb decrease; p53 and p21 increase by immunoblot/qPCR/immunofluorescence; senescence‐pathway transcriptomics	Sequential centrifugation and 110,000 × *g* ultracentrifugation; electron microscopy, NTA and CD63/TSG101/HSP70 immunoblotting	EVs	GPX4	Yes—donor GPX4 knockdown, GPX4 transfer tracing, pharmacological GPX4 inhibition and ferroptosis‐rescue experiments	senescent endothelial‐cell EVs transferred GPX4 to colorectal‐cancer cells, inhibited ferroptosis and promoted 5‐FU/oxaliplatin resistance	Yes—EV‐transfer xenograft and GPX4/chemotherapy intervention experiments; supportive patient EV analysis

*Notes*: EV terminology follows the original studies. Senescence validation summarizes the combination of markers reported in each study. Cargo‐specific causality was classified as ‘Yes’ only when depletion, overexpression, neutralization, pharmacological blockade, or rescue experiments linked a defined EV cargo to the phenotype; demonstration of EV dependence alone was not considered cargo‐specific causality. In vivo validation refers to validation of the reported EV‐dependent biological effect rather than detection of senescence or EVs alone. Rubicon–WIPI2d–ESCRT evidence is discussed in the text as contextual evidence and is not included as a therapy‐induced senEV study.

Abbreviations: APC, antigen‐presenting cell; CRC, colorectal cancer; DPP4, dipeptidyl peptidase 4; EGF, epidermal growth factor; GPX4, glutathione peroxidase 4; HUVEC, human umbilical vein endothelial cell; NTA, nanoparticle tracking analysis; PMN‐MDSC, polymorphonuclear myeloid‐derived suppressor cell; SA‐EV, senescence‐associated extracellular vesicle; sEV, small extracellular vesicle.

## 3 Mechanisms of senEV Generation and Cargo Loading

Direct senEV‐specific studies support increased EV release through DNA damage–p53‐associated and ceramide/nSMase2‐dependent mechanisms. By contrast, ESCRT‐mediated sorting, plasma‐membrane budding, and coupling to autophagy or mitochondrial quality control are supported mainly by broader EV studies and remain candidate mechanisms in senescent cells. The Rubicon–WIPI2d–ESCRT axis provides an additional ageing‐ and senescence‐associated mechanism that should be tested directly in TIS. Figure 2 distinguishes these evidence levels.

### 3.1 Why Is EV Secretion Increased in Senescent Cells? Stress Adaptation and Intercellular Communication

Takasugi et al. (2017) reported increased small EV secretion by senescent cells (Takasugi et al. 2017), whereas Hitomi et al. (2020) showed that DNA damage enhances senescence‐associated EV release through the ceramide pathway (Hitomi et al. 2020). Hitomi et al. further demonstrated that this release facilitates the removal of cytoplasmic DNA and limits excessive inflammatory responses (Hitomi et al. 2020). However, this protective effect in donor senescent cells does not imply that extracellular DNA is uniformly anti‐inflammatory, because EV‐associated DNA can activate innate immune signalling after uptake by recipient cells and may promote immunosuppressive responses in specific contexts (Lai et al. 2025; Dou et al. 2017; Gluck et al. 2017). These findings suggest that increased senEV secretion is not merely a passive by‐product of SASP activation but may contribute to cellular stress disposal and homeostatic adaptation (Figure 2). Senescent cells face multiple burdens—persistent DNA damage, accumulation of oxidized proteins/lipids and metabolic by‐products, and increased pressure on lysosomal and mitochondrial quality control. When conventional degradative pathways (such as the autophagy–lysosome system) are constrained or reprogrammed, cells may divert a portion of molecular or organellar components that are ‘difficult to degrade or unsuitable to retain’ into secretory pathways, thereby achieving particulate export through EVs (Gade et al. 2025; Sheng et al. 2025; Choezom and Gross 2022). This export may function as a stress‐adaptive and intercellular‐communication route in selected models: it may support intracellular homeostasis while also externalizing stress signals that reshape the microenvironment and immune responses (Tanaka and Takahashi 2021; Misawa et al. 2020; Urbanelli et al. 2016). This possibility provides a testable explanation for the context‐dependent effects of senEVs (Song et al. 2023; Ziglari et al. 2025; Almeida and Melo 2025; Liu et al. 2024; Zhang et al. 2024).

From a systems perspective, the secretory system of senescent cells contains at least two parallel channels: (a) soluble SASP, comprising cytokines, chemokines, proteases and related freely diffusible factors (van Deursen 2014; Kuilman et al. 2008; Rodier et al. 2009; Baker et al. 2011); and (b) particulate SASP, comprising EV‐associated signals whose membrane enclosure can support cargo protection, combined delivery and recipient‐cell uptake (Welsh et al. 2024; Thery et al. 2018; van Niel et al. 2018; Colombo et al. 2014; Kalluri and LeBleu 2020).

Under persistent damage, the advantage of particulate SASP is that it allows cells to dispose of and propagate signals in a more selective manner, outputting not only immune‐alerting cues but also immune‐suppressive or tolerance‐promoting cues (Tanaka and Takahashi 2021; Misawa et al. 2020; Urbanelli et al. 2016; Kalluri and McAndrews 2023). Understanding this point is important for the later discussion of which upstream signals may influence EV abundance and cargo loading (Welsh et al. 2024; Thery et al. 2018; van Niel et al. 2018; Colombo et al. 2014; Kalluri and LeBleu 2020).

### 3.2 The DDR→Ceramide/nSMase2 Axis: One of the Most Translatable Switches of Biogenesis

Among the upstream regulatory mechanisms proposed so far, the coupling between the DNA damage response (DDR) and the sphingolipid metabolism/sphingomyelinase axis provides one of the clearest and most potentially druggable explanations for why senescence increases senEV secretion (Choezom and Gross 2022; Hitomi et al. 2020; Menck et al. 2017; Pacifico et al. 2024). The core logic may be summarized as follows: DNA damage does not only trigger cell‐cycle arrest and transcriptional remodelling in the nucleus; through membrane lipid metabolic reprogramming, it also alters endosomal membrane dynamics and the probability of vesicle budding, thereby increasing EV release (Choezom and Gross 2022; Hitomi et al. 2020; Menck et al. 2017; Pacifico et al. 2024). DNA damage has been shown to downregulate sphingomyelin synthase 2 (SMS2), upregulate neutral sphingomyelinase 2 (nSMase2), activate ceramide signalling and increase senEV biogenesis (Hitomi et al. 2020).

At the mechanistic level, ceramide is considered an important lipid molecule that promotes membrane curvature changes and microdomain formation. It can drive inward budding of endosomal membranes to form ILVs (intraluminal vesicles), thereby providing a structural basis for the generation of exosome‐like sEVs (Hitomi et al. 2020; Trajkovic et al. 2008). Neutral sphingomyelinase 2 (nSMase2) hydrolyzes sphingomyelin to generate ceramide and is therefore regarded as a key molecular link between the DDR and senEV release (Choezom and Gross 2022; Hitomi et al. 2020; Menck et al. 2017; Trajkovic et al. 2008). Further studies have indicated that nSMase2 not only promotes membrane budding by providing ceramide, but may also influence the fate choice of MVBs by regulating endosomal acidification: when MVBs are more inclined to fuse with the plasma membrane rather than enter the lysosomal degradation pathway, secretory output is enhanced and sEV release correspondingly increases (Hitomi et al. 2020). Choezom and Gross (2022) demonstrated that nSMase2 counteracts vacuolar H+‐ATPase (V‐ATPase)‐mediated acidification of MVBs, thereby shifting their fate toward EV secretion rather than lysosomal degradation (Choezom and Gross 2022).

First, the DDR–ceramide–nSMase2 axis does more than increase senEV secretion; it may also participate in negative‐feedback mechanisms that limit excessive inflammation or cell death, suggesting that senEV release can function as a homeostatic protective response in certain contexts (Hitomi et al. 2020). Second, DDR does not regulate EVs only through nSMase2: the p53 axis has also been shown to regulate exosome secretion under stress through its target gene TSAP6/Steap3, constituting another interpretable ‘DDR–p53–secretory pathway’ chain (Wan et al. 2012; Burdakov et al. 2018; Ostrowski et al. 2010; Yu et al. 2006, 2009). Yu et al. (2006) and Lespagnol et al. (2008) demonstrated that: the p53 response can enhance exosome production, and the DNA damage‐induced p53‐dependent secretory pathway is markedly impaired when TSAP6/Steap3 is absent (Yu et al. 2006; Lespagnol et al. 2008). This means that, even when EV increase is similarly driven by DNA damage, the upstream modules may differ according to cell type and damage spectrum, thereby influencing cargo profiles and immune effects. Consistent with these stress‐response studies, Lehmann et al. demonstrated that ionizing radiation induced premature senescence and increased the release of exosome‐like EVs from human prostate cancer cells. Disruption of p53 signalling attenuated both senescence induction and vesicle release, providing direct evidence for a DNA damage response–p53‐associated increase in EV output in a TIS model. However, the downstream biological functions and cargo‐specific effects of the released vesicles were not examined in that study (Lehmann et al. 2008).

### 3.3 The Endosome–Multivesicular Body–Exosome Biogenesis Framework: How Senescence Alters ‘Endocytic–Secretory Flux’ and Cargo Loading

The following endosome–MVB framework is established primarily from general EV studies and is used here as mechanistic context; direct validation of each step in senescent cells remains limited. From the perspective of cell biology, the core biogenesis pathway of exosome‐like sEVs is as follows: plasma membrane internalization forms early endosomes → endosomes mature and form MVBs → MVBs fuse with the plasma membrane and release ILVs extracellularly (at which point ILVs are referred to as exosomes/part of sEVs) (van Niel et al. 2018; Colombo et al. 2014; Kalluri and LeBleu 2020; Ostrowski et al. 2010; Savina et al. 2002, 2005; Shen et al. 2011; van Niel et al. 2011; White et al. 2006). Two types of key decisions exist in this process: (a)how ILVs are formed and which cargos are loaded (membrane budding and cargo sorting); (b)whether MVBs ultimately proceed toward secretion or degradation (fusion with the plasma membrane vs. fusion with lysosomes).

Classic EV reviews have summarized multiple modules involved in ILV formation and cargo sorting, including ESCRT‐dependent and ESCRT‐independent mechanisms, lipid raft/sphingolipid‐related mechanisms, and sorting mechanisms involving membrane protein microdomains such as tetraspanins (Ostrowski et al. 2010; Savina et al. 2002, 2005; Shen et al. 2011; van Niel et al. 2011; White et al. 2006); At the level of MVB transport and fusion, Rab family small GTPases, the cytoskeleton, and membrane fusion modules together determine the efficiency of vesicle trafficking and membrane fusion. Among these regulators, Rab27a and Rab27b act at distinct steps of the exosome secretion pathway (Ostrowski et al. 2010), whereas the syndecan–syntenin–ALG‐2‐interacting protein X (ALIX) axis contributes to exosome formation and intraluminal budding within endosomes (Baietti et al. 2012). Because most of these modules were originally defined in non‐senescent cells, their application to senEV biogenesis should be regarded as contextual evidence unless directly validated in a senescence model. Yanagawa et al. identified the Rubicon–WIPI2d axis as an additional regulator of exosome biogenesis in ageing‐ and senescence‐associated systems. Although Rubicon is best known as a negative regulator of autophagy, its effect on EV production was not merely secondary to autophagy inhibition. Rubicon recruited WIPI2d to endosomes, where WIPI2d interacted with ESCRT components involved in intraluminal‐vesicle formation. Rubicon was also required for the age‐associated increase in exosome release and influenced exosomal microRNAs associated with senescence and longevity. These findings identify a non‐canonical Rubicon–WIPI2d–ESCRT pathway that should be tested directly in TIS; its contribution to cancer‐associated senEV biogenesis has not yet been established (Yanagawa et al. 2024).

Considering this general EV‐biogenesis framework in the context of cellular senescence leads to the hypothesis that senescence may reshape endocytic–secretory flux rather than merely altering a single cargo‐sorting protein. For example, senescent cells are often accompanied by increased cell size, elevated lysosomal/endosomal burden and changes in acidification/degradative capacity. In this context, MVBs that would originally be more likely to be sent to lysosomes for degradation may instead be more frequently redirected toward secretory release, thereby increasing total sEV output (Choezom and Gross 2022; Hitomi et al. 2020; Menck et al. 2017; Pacifico et al. 2024). In addition, senescence‐associated transcriptional programs (including p53‐related modules) may indirectly alter the probability of MVB secretion and cargo‐loading preference by affecting key nodes in membrane trafficking (Trajkovic et al. 2008; Wan et al. 2012; Burdakov et al. 2018; Yu et al. 2006, 2009). In other words, the increase in EVs in the senescent state may result both from increased production and from pathway rewiring from degradation to secretion (Choezom and Gross 2022; Hitomi et al. 2020; Menck et al. 2017; Pacifico et al. 2024).

Therefore, when discussing the mechanisms of senEV biogenesis, a more operational research question is: (a)in a given senescence model, does EV increase mainly result from enhanced ILV formation, or from MVB fate being biased toward secretion? (b)do cargo changes mainly result from altered sorting mechanisms, or from changes in the intracellular pool of materials awaiting disposal (for example, accumulation of damaged DNA or mitochondrial components)?

These questions directly determine the choice of later intervention points: if the major driver is the nSMase2/ceramide‐dependent membrane budding process, lipid‐metabolism targeting is more relevant; if the major driver is altered endosomal trafficking/lysosomal fate, then Rab/acidification/fusion‐related modules as well as lysosomal functional status need closer attention (Choezom and Gross 2022; Hitomi et al. 2020; Menck et al. 2017; Pacifico et al. 2024; Ostrowski et al. 2010; Lespagnol et al. 2008; Savina et al. 2002, 2005; Shen et al. 2011; van Niel et al. 2011; White et al. 2006).

### 3.4 Coupling of senEV Secretion With Autophagy and Mitochondrial Quality Control: From Intracellular Degradation to Secretory Clearance

Autophagy normally mediates the degradation of cytoplasmic protein aggregates and damaged organelles. However, under prolonged stress and functional reprogramming, autophagic pathways may exhibit insufficient capacity or altered flux, leading to the accumulation of damaged mitochondria, oxidatively damaged components and cytoplasmic DNA fragments (Carles‐Fontana et al. 2022; Gade et al. 2025; Iorio et al. 2024; Sheng et al. 2025; Dou et al. 2017; Gluck et al. 2017; Li et al. 2024; Choezom and Gross 2022). Under these conditions, some material that would otherwise undergo lysosomal degradation may instead be diverted into EV‐mediated secretion (Gade et al. 2025; Sheng et al. 2025; Choezom and Gross 2022). Unlike soluble secretory pathways, EVs can enclose membrane‐associated, aggregated, or organelle‐derived cargo within a lipid bilayer, thereby facilitating the export of material that cannot readily be released as freely soluble molecules. EV‐mediated export should therefore be regarded as a complementary route for the disposal of selected intracellular cargo rather than as a universally superior secretory pathway.

From this perspective, EV‐associated export can have two non‐mutually exclusive consequences: (a)EV‐mediated export reduces the retention of damaged mitochondria/oxidative products/nucleic acid fragments in the cytoplasm, thereby relieving stress and maintaining survival (Gade et al. 2025; Sheng et al. 2025); (b)microenvironmental signalling level: exported mitochondrial components or DNA/mtDNA may be read by neighbouring cells or immune cells as DAMP‐like signals, thereby translating an ‘intracellular crisis’ into a ‘tissue‐level immune/inflammatory program’ (Carles‐Fontana et al. 2022; Gade et al. 2025; Iorio et al. 2024; Sheng et al. 2025; Dou et al. 2017; Gluck et al. 2017; Li et al. 2024).

Secretory mitophagy refers here to a non‐canonical mitochondrial quality‐control process in which damaged mitochondrial components are exported from cells through an EV‐associated secretory pathway rather than being eliminated exclusively through lysosomal degradation (Gade et al. 2025). In this process, EVs, rather than mitophagy itself, serve as the carriers of mitochondrial cargo. Gade et al. reported that, under oxidative stress, cancer cells can bypass lysosomal degradation and export damaged mitochondrial material through EVs, a process termed secretory mitophagy (Gade et al. 2025). Iorio et al. subsequently summarized the major types, characteristics, and proposed biogenetic mechanisms of EVs carrying mitochondrial components (Iorio et al. 2024). In senescent tumour and stromal cells, as well as in cells exposed to therapeutic stress, this form of secretory clearance may support adaptation to oxidative stress while also releasing mitochondrial components that influence downstream immune responses (Carles‐Fontana et al. 2022; Gade et al. 2025; Lai et al. 2025; Sheng et al. 2025; Li et al. 2024). However, direct evidence that secretory mitophagy is broadly activated in validated senescent tumour or stromal cells remains limited, and its contribution to senEV biology should therefore be regarded as a hypothesis requiring further validation.

### 3.5 The Possible Contribution of Plasma Membrane‐Budding EVs (Microvesicles/Ectosomes) in Senescence: membrane Tension, Rho‐GTPases and ESCRT‐III

For clarity, endosomes are intracellular membrane‐bound compartments of the endocytic pathway. Exosomes are EVs of demonstrated endosomal origin that are generated as intraluminal vesicles within multivesicular endosomes and released when these endosomes fuse with the plasma membrane. Ectosomes, commonly referred to as microvesicles, are EVs generated by outward budding and fission of the plasma membrane. Consistent with MISEV2023, when the biogenetic origin of an EV population has not been experimentally demonstrated, operational terms such as small extracellular vesicles (sEVs) should be used rather than assigning an exosomal or ectosomal origin (Welsh et al. 2024; Thery et al. 2018; van Niel et al. 2018; Colombo et al. 2014). In addition to EVs of endosomal origin, senescent cells may also exhibit increased direct budding and release of ectosomes/microvesicles from the plasma membrane (Ratajczak et al. 2006; Muralidharan‐Chari et al. 2009, 2010; Wilson et al. 2004). This type of EV relies more heavily on plasma membrane–cortical cytoskeleton remodelling and changes in membrane lipid asymmetry, and its formation commonly involves small GTPase signalling, membrane lipid metabolism, myosin light‐chain phosphorylation, and relaxation of the membrane skeleton (Muralidharan‐Chari et al. 2009, 2010; Wilson et al. 2004). Among the classic studies, work on ARF6‐regulated shedding showed that the ARF6 GTP/GDP cycle can regulate plasma membrane‐derived microvesicle release through the phospholipase D–ERK–MLCK axis. Within a senescence‐related cellular context, increased cell size, altered membrane tension, and cytoskeletal remodelling may increase the likelihood of plasma membrane budding. We therefore propose that these changes could contribute to increased ectosome/microvesicle release, although this possibility requires direct experimental validation.

It is worth noting that ESCRT does not function only in inward budding within endosomes. During ectosome/microvesicle biogenesis, arrestin domain‐containing protein 1 (ARRDC1) recruits tumour susceptibility gene 101 protein (TSG101), a component of ESCRT‐I, through its Pro‐Ser‐Ala‐Pro (PSAP) motif, thereby promoting plasma membrane budding and scission and generating ARRDC1‐mediated microvesicles (Nabhan et al. 2012). Although this mechanism has not been specifically demonstrated as a senescence‐dependent pathway, it provides a testable mechanism through which senescence‐associated changes in ESCRT activity could influence plasma membrane budding. This suggests that senescence‐related changes in ESCRT availability/localization may simultaneously affect both the ‘endosomal budding’ and ‘plasma membrane budding’ pathways.

### 3.6 The ‘Selective Rules’ of Cargo Loading: Ubiquitination–ESCRT, the Glycocalyx–Syntenin Axis and RNA‐Binding Proteins–Sequence Motifs

The reason that senEVs can exert potent paracrine effects lies not only in their increased abundance but also in systematic changes in cargo composition (Misawa et al. 2020; Kalluri and McAndrews 2023). In broader EV systems, membrane proteins can enter ILVs through ESCRT‐related sorting, whereas the syndecan–syntenin–ALIX pathway may selectively enrich particular signalling complexes and membrane proteins (van Niel et al. 2018; Colombo et al. 2014; Kalluri and LeBleu 2020; Shen et al. 2011; van Niel et al. 2011; Kowal et al. 2016). Whether these mechanisms operate similarly in therapy‐induced senEVs requires direct testing. Under senescence/therapeutic stress, changes in membrane protein glycosylation/shedding and endocytic recycling may systemically drive specific ‘pro‐inflammatory/pro‐survival/immune‐regulatory’ cargos into EVs through these sorting axes.

With regard to RNA cargo, growing evidence suggests that loading is not ‘random’: specific RNA‐binding proteins (RBPs) can recognize RNA sequences or structural motifs and mediate the selective loading of these RNAs into EVs. For example, hnRNPA2B1 can recognize specific motifs in miRNAs, and its SUMOylation status affects miRNA loading efficiency. The microtubule‐associated protein 1 light chain 3 (LC3)‐dependent EV loading and secretion (LDELS) pathway provides an additional mechanism through which selected RNA‐binding proteins and RNAs can be incorporated into EVs. On this basis, we hypothesize that stress‐associated translational and RNA‐metabolic reprogramming in senescent cells may alter RNA‐binding protein modification and autophagy–secretion coupling, thereby selectively enriching miRNAs and RNA‐binding protein complexes that reinforce senescence‐associated signalling or prime recipient immune cells. This process may amplify paracrine senescence and immune remodelling, but the general RNA‐sorting mechanism remains contextual unless tested in senEV models. Borghesan et al. provided direct evidence that small EVs participate in paracrine senescence. Small EVs released by oncogene‐induced or DNA damage‐induced senescent fibroblasts induced a senescence‐associated transcriptional and growth‐arrest phenotype in recipient cells. Proteomic profiling followed by functional perturbation identified interferon‐induced transmembrane protein 3 (IFITM3) as a partially causal senEV cargo: depletion of IFITM3 in donor cells attenuated, whereas enrichment of IFITM3 in EVs promoted, recipient‐cell senescence. This study therefore represents senEV‐specific evidence for EV‐mediated intercellular propagation of senescence rather than an extrapolation from general EV biology (Borghesan et al. 2019)

The selective‐loading process described above determines not only the molecular composition of senEVs but also the stress‐associated signals delivered to recipient immune cells. Especially under therapeutic stress where mitochondrial dysfunction, elevated ROS and nucleic acid leakage coexist, whether preferential loading of mitochondrial components or mtDNA contributes to divergent immune outcomes is therefore a testable candidate explanation rather than an established dividing line. On this basis, the next section will specifically discuss the mechanistic links among mitochondrial stress, mtDNA export and EV‐mediated immune remodelling. Because existing studies differ substantially in senescence inducer, donor‐cell type, sampling time and EV isolation method, direct comparisons across senescence models remain limited; representative studies are summarized in Table 1.

## 4 Mitochondrial Stress and EV Cargo: Direct Evidence and Emerging Hypotheses

Direct experimental evidence links EV‐associated mtDNA released by senescent tumour cells to PMN‐MDSC‐mediated immunosuppression (Lai et al. 2025). On this basis, we propose mitochondrial stress as a candidate, rather than established, upstream determinant of senEV immune directionality (Figure 3).

### 4.1 Mitochondrial and Nucleic Acid Stress as Candidate Regulators of senEV Immune Effects

The preceding section summarized candidate mechanisms that may contribute to senEV biogenesis, including intraluminal vesicle formation within MVBs, endosomal trafficking and release, plasma membrane budding of ectosomes/microvesicles, and selective cargo loading. Because direct evidence in senescent cells remains limited, these pathways should be regarded as candidate mechanisms rather than fully established senEV‐specific biogenesis routes. Building on this framework, a more integrative question is why EVs generated under apparently similar therapeutic or senescence‐associated conditions can produce divergent immune outcomes. The key may not lie in whether EVs are present, but rather in what type and what intensity of stress information they carry. Under conditions of aggravated mitochondrial stress, cells are more prone to elevated ROS, altered membrane permeability, abnormal exposure of mitochondrial components, and nucleic acid leakage, and may package some of these danger‐ or stress‐associated cues into EVs for delivery to immune cells and tumour–stromal cells, thereby resetting the innate immune threshold and inflammatory response mode of recipient cells (Carles‐Fontana et al. 2022; Gade et al. 2025; Lai et al. 2025; Iorio et al. 2024; Sheng et al. 2025; Dou et al. 2017; Gluck et al. 2017; Li et al. 2024). These observations raise the possibility that mitochondrial stress may shape the downstream immune consequences of senEV‐mediated communication.

### 4.2 Mitochondrial Signalling: From Senescence‐Associated Mitochondrial Dysfunction to EV‐Associated Mitochondrial Cargo and Immune Outcomes

Here, senescence‐associated mitochondrial dysfunction refers to impaired mitochondrial quality control and the accumulation of mitochondrial damage in senescent cells, including altered bioenergetics, elevated ROS levels, loss of mitochondrial membrane integrity and mtDNA leakage. These changes may promote the incorporation of mitochondrial components into senEVs and thereby influence downstream immune responses (Carles‐Fontana et al. 2022; Gade et al. 2025; Lai et al. 2025; Iorio et al. 2024; Sheng et al. 2025; Li et al. 2024).

Terminology follows the operational definitions provided in the Introduction. Study‐specific terms such as ‘mitochondria‐encapsulating EVs,’ ‘mitochondrial cargo‐containing EVs,’ and ‘EV‐Mito’ are retained only when describing the corresponding source studies.

Senescence and therapeutic stress are frequently accompanied by mitochondrial dysfunction, elevated ROS levels and impaired membrane homeostasis. Because ROS are short‐lived, it is the oxidative damage caused by sustained ROS production, rather than ROS themselves, that accumulates. Such damage to mitochondrial proteins, lipids, and DNA may increase the likelihood of mtDNA leakage and the persistence of damaged mitochondrial fragments. Studies in broader, non‐senescent stress models of mitoEVs, mitochondria‐encapsulating EVs, and secretory mitophagy suggest that, when mitochondrial quality‐control pressure becomes excessive or lysosomal degradation is constrained, cells can export mitochondrial components through EV‐associated pathways (Gade et al. 2025; Iorio et al. 2024; Sheng et al. 2025; Li et al. 2024; Wang et al. 2025). Whether the same process is consistently activated in therapy‐induced senescent cells remains to be established. Although contamination remains a technical concern, several studies support the idea that mitochondrial cargo loading can occur in an organized manner under stress conditions. Except for EV‐associated mtDNA transfer directly demonstrated in senescent‐cell models (Lai et al. 2025), these mitochondrial‐EV mechanisms have largely been defined outside the context of cellular senescence and are therefore discussed here as contextual evidence.

The key significance of this process is that it ‘packages and externalizes’ intracellular nucleic acid and mitochondrial stress signals (Carles‐Fontana et al. 2022; Gade et al. 2025; Iorio et al. 2024; Sheng et al. 2025; Dou et al. 2017; Gluck et al. 2017; Li et al. 2024). When EVs containing mtDNA or other mitochondria‐related molecules are taken up by recipient immune cells, including dendritic cells, macrophages and PMN‐MDSCs, the transferred DNA may engage the cGAS–STING pathway in a recipient‐cell‐dependent manner, as discussed in Sections 4.5 and 4.6. An unresolved question is whether impaired mitochondrial quality control preferentially drives degradation or extracellular extrusion pathways.

### 4.3 CGAS–STING and ‘Abnormal Nuclear/Cytoplasmic DNA’ as the Core of Senescence‐Associated Inflammation

An important axis in senescence‐associated inflammation is the recognition of abnormal cytoplasmic DNA by cGAS–STING, which can contribute to inflammatory transcriptional programs and paracrine senescence (Dou et al. 2017; Gluck et al. 2017). Classic studies have shown that cytoplasmic chromatin fragments (CCFs) can form during senescence and, once recognized by cGAS, trigger STING‐related signalling, thereby amplifying inflammation and SASP output (Dou et al. 2017; Gluck et al. 2017). This indicates that DNA sensing is not merely a downstream accompanying phenomenon of senescence, but rather an important mechanistic support for maintaining its inflammatory phenotype.

When this axis is considered in the EV context, it is important to distinguish intercellular DNA transfer from intracellular DNA sensing. Senescent donor cells may externalize DNA or mtDNA by packaging it into EVs, but they do not externalize the DNA‐sensing machinery itself. After EV uptake and cytosolic release of the DNA cargo, the endogenous cGAS–STING pathway of the recipient cell may be activated. Thus, the DNA ligand is transferred between cells, whereas DNA sensing occurs de novo within the recipient cell. Therefore, senEVs are not only a particulate form of SASP, but may also serve as carriers for the propagation of DNA‐sensing signals. The key question is no longer simply whether they carry nucleic acids, but rather who takes up those nucleic acids, when and where they are recognized, and what kind of immune program is ultimately induced.

### 4.4 Secretory Mitophagy: A Candidate Bridge Between Mitochondrial Quality Control and Immune Signalling

In the classical view, damaged mitochondria are mainly degraded through mitophagy after delivery to lysosomes. However, secretory mitophagy proposes a supplementary model that is better suited to explaining conditions of sustained therapeutic stress: when the mitochondrial damage burden is too high, or when autophagy–lysosome flux is insufficient, cells may carry out ‘secretory clearance’ by exporting some damaged mitochondria in EV form, thereby relieving intracellular stress and ensuring survival (Gade et al. 2025; Sheng et al. 2025). These observations suggest that mitochondrial quality control and EV biogenesis may become coupled in senescent cells rather than operating as entirely independent pathways.

When this concept is brought back into the TIS/senescence context, its theoretical importance lies in providing an interpretable upstream mechanism for why senescent cells release more mitochondria‐related cargo (Gade et al. 2025; Sheng et al. 2025). More importantly, it links mitochondrial quality control to immune regulation: (a) For donor cells, this is a form of ‘detox‐like secretion’ that favours homeostasis and survival (Gade et al. 2025); (b) For tissues/the immune system, it amounts to releasing damaged mitochondria and nucleic acids as potential DAMP‐like signals into the extracellular space, making them available as sources of stimulation—or tolerance—for immune cells to interpret (Carles‐Fontana et al. 2022; Gade et al. 2025; Iorio et al. 2024; Sheng et al. 2025; Dou et al. 2017; Gluck et al. 2017; Li et al. 2024).

Accordingly, secretory mitophagy provides a plausible link between mitochondrial stress and EV‐associated immune signalling, although it has not yet been established as a dominant pathway in therapy‐induced senescent cells.

### 4.5 MtDNA/Cytoplasmic DNA → cGAS–STING: EVs ‘Externalize’ the DNA‐Sensing Axis, but the Direction Depends on the Recipient and the Time Window

As discussed in Section 4.3, cGAS–STING is an important endogenous axis of senescence‐associated inflammation; when EVs carry DNA/mtDNA and are taken up by immune cells, this axis may be re‐triggered in recipient cells (Dou et al. 2017; Gluck et al. 2017) (Figure 3). Mechanistically, cGAS activation in recipient cells can engage STING‐dependent TBK1–IRF3/type I interferon and NF‐κB signalling, with the resulting immune effect depending on the recipient‐cell population and the duration of stimulation (Lai et al. 2025; Dou et al. 2017; Gluck et al. 2017). In broader mtDNA and EV systems, type I interferon‐associated responses in antigen‐presenting cells may enhance maturation, antigen presentation, and effector T‐cell priming. However, this complete pathway has not yet been directly demonstrated using EVs from experimentally validated senescent‐cell models. SenEV‐dependent recruitment of MHC‐II‐positive antigen‐presenting cells and antitumour immune surveillance has been demonstrated experimentally (Ziglari et al. 2025; Almeida and Melo 2025), although mtDNA has not been established as the responsible senEV cargo in this model. In contrast, direct evidence shows that mtDNA‐containing EVs released by senescent tumour cells activate cGAS–STING–NF‐κB signalling in PMN‐MDSCs and enhance immunosuppression (Lai et al. 2025).

Therefore, EV‐associated mtDNA is neither inherently antitumour nor inherently protumour; it should be considered a context‐dependent signal whose consequences depend on recipient‐cell identity, dose and signalling state (Carles‐Fontana et al. 2022; Gade et al. 2025; Iorio et al. 2024; Sheng et al. 2025; Dou et al. 2017; Gluck et al. 2017; Li et al. 2024).

### 4.6 A Key Closed Loop of Evidence: mtDNA Signals Released by Senescent Tumour Cells Enhance PMN‐MDSC‐Mediated Immunosuppression

Direct evidence shows that senescent tumour cells package extracellular mtDNA within EVs and selectively transfer it to PMN‐MDSCs, where cGAS–STING–NF‐κB signalling enhances immunosuppressive activity and promotes tumour progression (Lai et al. 2025).

PMN‐MDSC‐mediated immunosuppression reduces antitumour T‐cell activity and weakens immune surveillance, thereby enabling tumour cells to evade immune elimination. In this context, immune escape is not a separate or contradictory phenomenon but a downstream consequence of the immunosuppressive state.

This mechanistic sequence is particularly relevant to the post‐treatment tumour microenvironment, in which senescent residual cells may persist, myeloid populations may expand or undergo immunosuppressive reprogramming, and the surrounding immune niche may limit responses to immunotherapy (Dong et al. 2024; Song et al. 2023; Ding et al. 2025; Liu et al. 2024; Lai et al. 2025; Zhang et al. 2024; Kalluri and McAndrews 2023; Ahn et al. 2025; Kuang et al. 2025; Spada and Ganguly 2025; Yue et al. 2023; Saleh 2024; Wang et al. 2025; Xiao et al. 2023). Although this study directly demonstrated EV‐mediated mtDNA transfer from senescent cells, the responsible EV subtype, cargo‐loading mechanism, and generalizability across different tumour and senescence models remain to be established.

From a translational perspective, these findings support further investigation of whether selectively targeting mtDNA loading, release, uptake, or sensing could be more precise than globally inhibiting all EV populations.

### 4.7 Mitochondrial Cargo‐Containing EVs: Metabolic Transfer and Potential Immune Relevance

Evidence from non‐senescent models indicates that EVs containing mitochondrial material can transfer functional mitochondrial components to recipient cells (Wang et al. 2025). In mesenchymal stem cells, the release of mitochondria‐containing extracellular vesicles (EV‐Mito) is regulated by a calcium‐dependent CD38–IP3R pathway (Wang et al. 2025). However, this study did not examine TIS or antitumour immunity, and a direct relationship among mitochondria‐enriched senEVs, ROS, and PD‐L1‐mediated immune escape has not yet been established.

These observations suggest several testable intervention points rather than established therapeutic targets: (a) **EV biogenesis/loading nodes**: limiting the entry of mitochondrial cargo into EVs or restricting the release of particular EV subtypes (Carles‐Fontana et al. 2022; Gade et al. 2025; Iorio et al. 2024; Sheng et al. 2025; Li et al. 2024); (b) **ROS and metabolic nodes**: reducing mitochondrial stress and ROS‐driven signalling at the source, thereby decreasing the output of ‘immunosuppressive mitochondrial information’ (Carles‐Fontana et al. 2022; Gade et al. 2025; Iorio et al. 2024; Sheng et al. 2025; Iorio et al. 2024; Sheng et al. 2025; Li et al. 2024); (c) **Checkpoint nodes**: combining these approaches with PD‐1/PD‐L1 blockade in immunotherapy to test whether mitochondrial cargo‐containing EVs are upstream drivers of resistance (Kalluri and McAndrews 2023; Ahn et al. 2025; Kuang et al. 2025; Spada and Ganguly 2025; Yue et al. 2023).

Based on the evidence summarized above, we propose a cargo‐guided, node‐specific intervention framework. Candidate pathogenic cargos—such as mtDNA, mitochondrial proteins or metabolites, or intact mitochondrial structures reported in specific models—should first be identified and causally validated. The corresponding cargo‐loading, EV‐release, cellular‐uptake, or recipient‐cell‐sensing pathway could then be selectively targeted while preserving the physiological functions of other EV populations.

### 4.8 Cancer‐Cell Metabolic Reprogramming: EV‐Mediated Mitochondrial Transfer/Functional Remodelling and Drug Resistance

From a broader tumour‐biology perspective, the significance of mitochondrial EV cargo is not limited to immunity; it also extends to post‐treatment adaptation and the evolution of drug resistance. EV‐mediated transfer of mitochondrial components may help residual tumour cells survive the immediate post‐treatment selection period, during which treatment‐induced damage, metabolic stress, and immune pressure eliminate a substantial proportion of tumour cells. However, the contribution of mitochondrial EV cargo to this process remains a hypothesis that requires direct experimental validation. Separately, Jia et al. showed that senescent endothelial cell‐derived EVs transfer glutathione peroxidase 4 (GPX4) and confer ferroptosis resistance and chemotherapy resistance. This is a directly demonstrated, cargo‐specific senEV mechanism, but it does not establish the transfer of intact mitochondria or a general mitoEV pathway (Jia et al. 2026).

What is being emphasized here is not the classical proliferative signalling pathway, but rather the transferability of more fundamental capacities related to energy metabolism and stress tolerance (Lai et al. 2025; Wang et al. 2025). Therefore, future studies of mitoEVs cannot stop at the descriptive level of merely detecting mtDNA or mitochondrial markers. They must go on to determine whether these cargos are membrane‐protected, whether they truly enter specific recipient cells, whether they remodel the metabolic state of those cells, and whether they ultimately alter therapeutic responses. Only by linking cargo detection, intracellular or extracellular localization, effects on recipient‐cell function, and disease outcomes can researchers determine whether mitochondrial EV cargo acts as a mechanistic mediator and whether it can be developed as a biomarker or therapeutic target.

### 4.9 Summary: Mitochondrial Signalling as a Working Hypothesis for senEV Directionality

In summary, mitochondrial stress provides a plausible framework linking senEV cargo composition to downstream immune outcomes. Direct evidence currently establishes that mtDNA‐containing EVs released by senescent tumour cells can activate cGAS–STING–NF‐κB signalling in PMN‐MDSCs and enhance immunosuppression (Lai et al. 2025). Whether mitochondrial stress more broadly determines senEV cargo selection and immune directionality across different cell types, treatment modalities and time windows remains a working hypothesis.

Within this working model, mitochondrial signalling may provide a mechanistic intermediate layer for the later discussion of the ‘double‐edged’ effects of senEVs in cancer therapy. It may help explain why EVs of similar origin and similar abundance may lead to different outcomes, and it also suggests that future translational interventions should not stop at ‘suppressing the total amount of EVs,’ but should instead move toward ‘identifying and intercepting pathogenic mitochondrial cargo profiles that have been causally validated in defined models’. On this basis, the next section returns this mechanistic framework to the context of cancer therapy and specifically discusses how senEVs can exhibit both tumour‐promoting and tumour‐suppressive effects after treatment.

## 5 The Double‐Edged Role of senEVs in Cancer Therapy: Tumour‐Promoting and Tumour‐Suppressive Mechanisms

In addition to inducing tumour‐cell death, cancer treatments such as radiotherapy, chemotherapy, and targeted therapy can cause surviving tumour or stromal cells to enter TIS, defined here as an irreversible growth arrest accompanied by characteristic senescence‐associated molecular and secretory changes (Dong et al. 2024; Song et al. 2023; Ding et al. 2025; Liu et al. 2024; Lai et al. 2025; Zhang et al. 2024; Saleh 2024; Wang et al. 2025; Xiao et al. 2023). In this review, the term ‘senescence‐like state’ is used only for treatment‐stressed cells that display some senescence‐associated features but for which irreversible growth arrest has not been demonstrated. These cells exert persistent effects on the microenvironment with SASP as the core, and EVs, as a form of ‘particulate SASP,’ are able to package and deliver complex cargos over long distances, making post‐therapy signal export no longer merely ‘diffusion of soluble factors,’ but rather a more structured and targetable network of particulate signalling (Wang et al. 2024; Tanaka and Takahashi 2021; Misawa et al. 2020; Urbanelli et al. 2016; Kalluri and McAndrews 2023). The key paradox is that senEVs can either promote immune‐mediated clearance and suppress recurrence or support residual tumour‐cell growth and immune escape, depending on their cargo and recipient‐cell populations (Ziglari et al. 2025; Lai et al. 2025; Zhang et al. 2024; Takasugi et al. 2017). Accordingly, this section presents the evidence chains for both tumour‐promoting and tumour‐suppressive effects within the same logical framework, and then summarizes candidate variables that may shape functional directionality (Figure 4).

Importantly, these divergent effects are not unique to senEVs. Soluble SASP factors can promote immune‐cell recruitment and senescent‐cell clearance in some contexts (Kang et al. 2011; Xue et al. 2007), whereas persistent cytokine and chemokine signalling can sustain chronic inflammation and paracrine senescence in others (Kuilman et al. 2008; Rodier et al. 2009). The distinguishing feature of senEVs is therefore not bidirectionality itself but their ability to package proteins, lipids, nucleic acids and mitochondrial components within membrane‐bound particles and deliver these cargos through vesicle uptake (Welsh et al. 2024; Kalluri and McAndrews 2023).

### 5.1 Tumour‐Promoting Example 1: senEVs Drive Regrowth of Residual Cells Through Pro‐Proliferative Signals Such as EphA2

In the tumour‐promoting direction, the most intuitive mechanism is that senEVs directly deliver pro‐proliferative/pro‐survival membrane proteins or signalling complexes to recipient tumour cells, thereby re‐igniting growth pathways in cell populations that, after treatment, ‘should have remained suppressed’ (Takasugi et al. 2017). Classic work has suggested that small EVs secreted by senescent cells can be enriched in EphA2, and through membrane receptor‐ligand interactions and downstream signalling cascades, activate proliferative programs in recipient cells, ultimately manifesting as enhanced tumour cell proliferation and the establishment of a growth advantage (Takasugi et al. 2017).

This type of mechanism deserves particular attention in the therapeutic context: after treatment, the tumour microenvironment often displays persistent DNA‐damage signalling, chronic inflammatory cues, nutrient limitation, and altered redox conditions, which may increase the responsiveness of residual tumour cells to extracellular signals. If senEVs provide a ‘sufficiently strong growth drive’ at this point, then even if the overall tumour burden decreases, a small number of residual cells may still be ‘restarted’ and become seeds of recurrence (Takasugi et al. 2017). Here, mechanism‐based therapeutic intervention refers to the selective disruption of a defined causal step in the senEV pathway, such as cargo loading in donor cells, EV release, uptake by recipient cells, or activation of downstream signalling. In this membrane protein‐driven tumour‐promoting pathway, candidate intervention points include the selective loading of pro‐tumourigenic membrane proteins in senescent donor cells (van Niel et al. 2018; Colombo et al. 2014; Kalluri and LeBleu 2020; Takasugi 2018; Kowal et al. 2016) and the uptake or membrane fusion of senEVs in recipient cells (van Niel et al. 2018; Colombo et al. 2014; Kalluri and LeBleu 2020). Another potentially relevant variable is the duration for which senEVs remain available within the tumour microenvironment. However, the extracellular residence time of senEVs has not been systematically characterized and should therefore be directly measured rather than assumed to be prolonged. These candidate intervention points provide testable targets for selectively blocking pathogenic senEV‐mediated cargo transfer while minimizing interference with the physiological functions of other EV populations.

### 5.2 Tumour‐Promoting Example 2: senEV‐Mediated NF‐κB Signalling in Tumour Cells and PMN‐MDSCs

Compared with the direct delivery of pro‐proliferative signals, another tumour‐promoting chain that is closer to clinical reality arises from senescence‐like tumour cells that remain after treatment: these cells are both products of therapeutic stress and participants in the evolution of recurrence. A colorectal cancer (CRC) example provides a more complete closed loop: Zhang et al. (2024) reported that therapy‐induced senescent colorectal cancer cells released increased numbers of EVs enriched in SERPINE1 (Zhang et al. 2024). After uptake by recipient colorectal cancer cells, EV‐associated SERPINE1 bound to NF‐κB p65 and promoted its nuclear translocation (Zhang et al. 2024). Nuclear translocation of p65 is a signalling event, rather than a transcriptional event, that enables p65 to regulate NF‐κB‐dependent gene transcription. Activation of this pathway was associated with increased colorectal cancer cell proliferation, migration and invasion, together with decreased E‐cadherin expression and increased expression of Snail, Slug and MMP‐9 (Zhang et al. 2024).

The importance of this evidence chain is that it links ‘therapy—senescent residual cells—EVs—progression/recurrence risk’ into an interpretable causal pathway (Song et al. 2023; Zhang et al. 2024): (a) **Upstream trigger**: therapeutic stress drives a fraction of tumour cells into a TIS/senescence‐like state; (b) **Intermediate carrier**: the senEVs released by these cells undergo a shift in cargo loading; (c) **Downstream readout**: key inflammatory/survival pathways in recipient cells (such as NF‐κB) are re‐ignited, forming transcriptional programs that promote invasion, survival and microenvironmental remodelling.

NF‐κB signalling is located at a key regulatory node within the post‐treatment tumour microenvironment. Its activation can promote tumour‐cell survival and invasion and may also reinforce chronic inflammatory signalling through cytokine networks, thereby extending its effects from recipient tumour cells to the surrounding tumour niche (Dong et al. 2024; Wang et al. 2024; Zhang et al. 2024). Therefore, the SERPINE1–NF‐κB chain is not only one tumour‐promoting example, but also suggests that EVs from therapy‐induced senescent cells may be a key mediator that translates ‘treatment‐induced damage’ into ‘chronic tumour‐promoting inflammation and tissue remodelling’ (Song et al. 2023; Ding et al. 2025; Liu et al. 2024; Lai et al. 2025; Zhang et al. 2024; Saleh 2024; Wang et al. 2025; Xiao et al. 2023).

In addition to these direct effects on recipient tumour cells, senEVs can promote tumour progression through immune‐cell remodelling. Lai et al. demonstrated that mtDNA released within EVs by primary senescent cells and therapy‐induced senescent tumour cells was selectively transferred to PMN‐MDSCs, where it activated cGAS–STING–NF‐κB signalling, enhanced immunosuppressive activity and promoted tumour progression (Lai et al. 2025). Inhibition of VDAC‐dependent mtDNA release reduced PMN‐MDSC‐mediated immunosuppression and improved chemotherapy efficacy (Lai et al. 2025). This pathway provides a direct example of immune‐mediated tumour promotion and links the mitochondrial mechanism described in Section 4 to the functional outcomes discussed here.

### 5.3 Tumour‐Promoting Example 3: Senescent Endothelial Cell‐Derived EVs Transfer GPX4 and Promote Chemoresistance

Jia et al. demonstrated that 5‐fluorouracil‐ or oxaliplatin‐induced senescent endothelial cells accumulated glutathione peroxidase 4 (GPX4) and transferred this protein to colorectal cancer cells through EVs. The transferred GPX4 inhibited ferroptosis and increased resistance to 5‐fluorouracil and oxaliplatin. Donor‐cell GPX4 knockdown and pharmacological GPX4 inhibition attenuated the EV‐mediated phenotype, whereas in vivo EV‐transfer and treatment experiments supported the mechanism. This study extends senEV‐mediated tumour promotion from senescent cancer cells to the stromal compartment and provides cargo‐specific, in vivo‐supported evidence for therapy resistance (Jia et al. 2026).

### 5.4 Tumour‐Suppressive Example: senEVs Coordinate Immune Surveillance and Suppress Recurrence

Alongside these tumour‐promoting mechanisms, senEVs can also support immune‐mediated clearance of senescent tumour cells. Using an immunocompetent mouse model, Ziglari et al. demonstrated that senEVs released by chemotherapy‐treated senescent tumour cells were required for efficient senescent‐cell clearance and suppression of tumour recurrence (Ziglari et al. 2025). Blocking senEV release markedly impaired the recruitment of MHC‐II‐positive antigen‐presenting cells (APCs) and redirected the predominant response toward neutrophils. SenEV‐associated ligands also promoted APC–T‐cell adhesion and immune‐synapse formation, supporting a T‐cell‐mediated antitumour response (Ziglari et al. 2025; Almeida and Melo 2025).

These findings indicate that the tumour‐suppressive effect of senEVs in this model was mediated primarily through the recruitment of MHC‐II‐positive APCs and their interaction with T cells, rather than through an undefined form of ‘immune surveillance.’ However, the specific senEV cargos responsible for APC recruitment and the generalizability of this mechanism across tumour types and treatment conditions remain to be determined (Ziglari et al. 2025; Almeida and Melo 2025).

Almeida and Melo (2025) emphasized that the effects of senEVs depend on their cargo composition, the immune‐cell subsets that receive them, and the therapeutic and tissue contexts in which senEV‐mediated communication occurs (Almeida and Melo 2025). The existence of these questions itself indicates that research on senEVs must move toward a refined correspondence of ‘subtype—cargo—recipient cell—outcome,’ rather than remaining at the coarse‐grained level of simply describing ‘more EVs/fewer EVs’ (Tanaka and Takahashi 2021; Misawa et al. 2020; Urbanelli et al. 2016; Ziglari et al. 2025; Almeida and Melo 2025; Kalluri and McAndrews 2023).

### 5.5 Candidate Modifiers of senEV Function: Donor‐Cell Source, Treatment Type, Time After Treatment and Immune Context

Taken together, the available tumour‐promoting and tumour‐suppressive studies suggest a four‐variable working framework for organizing candidate modifiers of senEV function: donor‐cell source, treatment type, time after treatment and immune context (Ding et al. 2025; Xiao et al. 2023).

1) Source cells: Tumour cells versus stromal cells (such as CAFs) versus immune cells

Although all these vesicles can be classified as senEVs, their biological effects may differ according to their cellular origin. Tumour cell‐derived senEVs may directly influence tumour‐cell survival, invasion and metabolic adaptation. By contrast, stromal cell‐derived senEVs may modulate tumour behaviour indirectly through extracellular matrix remodelling, paracrine signalling, and immune‐cell priming or functional reprogramming. Cancer‐associated fibroblasts (CAFs) represent one important stromal source. Differences in cellular source may shape the starting cargo pool, the spectrum of recipient cells and the spatial distribution of senEV effects (Tanaka and Takahashi 2021; Misawa et al. 2020; Urbanelli et al. 2016; Kalluri and McAndrews 2023).

2) Treatment type: Different stress spectra induced by radiotherapy/chemotherapy/targeted therapy

Different treatments generate distinct combinations and intensities of DNA double‐strand breaks, replication stress, elevated ROS levels and mitochondrial dysfunction. These differences may alter the intracellular cargo pool and cargo‐sorting machinery of senescent cells, thereby changing the molecular composition of the senEVs they release (Choezom and Gross 2022; Hitomi et al. 2020; Menck et al. 2017; Pacifico et al. 2024). This also means that, even within the same tumour type, senEVs may move in entirely different functional directions under different treatment strategies.

3) Time after treatment: A candidate modifier requiring longitudinal validation

Time after treatment is a candidate modifier rather than an established directional switch. Clearance‐promoting and immunosuppressive senEV effects have been reported in different experimental models, but no longitudinal study has shown that a single senEV system reproducibly transitions from an early antitumour phase to a later protumour phase. The acute‐versus‐persistent distinction is therefore presented as a testable framework rather than a demonstrated general sequence.

4) Immune context: Immune‐hot versus immune‐cold states, MDSC burden and strength of immunosuppressive axes

Immune context may influence how senEV‐associated signals are interpreted. In immune‐infiltrated settings with effective antigen presentation, selected senEV signals may support surveillance, whereas in models with abundant MDSCs or dominant suppressive pathways, senEV‐associated mtDNA can reinforce immunosuppression (Lai et al. 2025). These possibilities have been demonstrated in different experimental systems and should not be interpreted as alternative outcomes of an otherwise identical senEV preparation.

## 6 Immune Regulation: Established senEV Activities and Conceptual Models of Antitumour Immunity

Section 5 summarized experimental examples showing that senEVs can either support immune‐mediated clearance or promote tumour growth and immunosuppression (Ziglari et al. 2025; Lai et al. 2025; Zhang et al. 2024; Takasugi et al. 2017). The purpose of this section is not to repeat these opposing outcomes but to examine the immune mechanisms and conceptual frameworks that may explain them. Direct senEV evidence is currently strongest for MHC‐II‐positive antigen‐presenting‐cell recruitment and senescent‐cell clearance (Ziglari et al. 2025), selected macrophage responses (Alibhai et al. 2020; Miyazoe et al. 2020) and mtDNA‐mediated PMN‐MDSC immunosuppression (Lai et al. 2025). By contrast, the temporal model, cancer‐immunity‐cycle mapping, and broader effects on T cells, NK cells, and immunotherapy responses remain working hypotheses based partly on general EV and tumour‐immunology evidence.

### 6.1 The Physiological/Pathological Duality of SASP: Immune Recruitment and Tissue Repair Versus Chronic Inflammation and Immunosuppression

The biological effects of the SASP are bidirectional and context dependent rather than intrinsically biphasic. Their direction is determined by the composition and concentration of SASP factors, the senescence inducer and duration, the identity of recipient cells, and the tissue and immune context. SASP components that recruit and activate immune cells can support senescent‐cell clearance and tissue repair, whereas persistent pro‐inflammatory, pro‐fibrotic, or immunosuppressive components can promote chronic inflammation, tissue remodelling, immune dysfunction and tumour progression. SenEVs exhibit similarly context‐dependent functional heterogeneity: depending on their cargo, they may support immune clearance or promote immune escape and metabolic adaptation (Wang et al. 2024; Ziglari et al. 2025; Almeida and Melo 2025).

Soluble SASP factors can also propagate senescence‐associated signals beyond donor cells through paracrine bystander effects. Nelson et al. (2012) showed that senescent cells induced a DNA damage response and secondary senescence in neighbouring fibroblasts through processes involving ROS and gap junction‐mediated cell‐cell communication (Nelson et al. 2012). In addition to soluble and gap‐junction‐mediated bystander signalling, Borghesan et al. demonstrated that small EVs released by senescent cells can transmit paracrine senescence, with IFITM3 accounting for part of the effect (Borghesan et al. 2019). SenEVs should therefore not be considered the only mediators of persistent or spatial signalling. Rather, they may complement soluble SASP factors by protecting selected cargo from extracellular degradation and co‐delivering multiple molecular signals to recipient cells.

### 6.2 Established Activity: senEV‐Mediated Recruitment of Antigen‐Presenting Cells and Senescence Surveillance

The concept of senescence surveillance holds that the immune system is not a passive bystander, but is capable of recognizing and clearing senescent or premalignant cells, thereby limiting tumour initiation and progression (Kang et al. 2011; Xue et al. 2007). Kang et al. (2011) demonstrated that CD4+ T‐cell‐dependent immune clearance of premalignant senescent hepatocytes limited liver cancer development, whereas Xue et al. (2007) showed that p53 restoration induced senescence and an innate immune response associated with the clearance of murine liver carcinomas (Kang et al. 2011; Xue et al. 2007).

Ziglari et al. (2025) demonstrated in an immunocompetent mouse model that senEVs were necessary and sufficient for the recruitment of MHC‐II‐positive antigen‐presenting cells and for immune‐mediated clearance of senescent cells (Ziglari et al. 2025). On the basis of this evidence, we hypothesize that senEVs may contribute to the spatial propagation of signals involved in senescence surveillance, although the signalling range and broader in vivo relevance of this process remain to be established. Soluble SASP factors can also transmit signals beyond donor cells and induce paracrine or bystander effects in neighbouring cells (Nelson et al. 2012). SenEVs should therefore be regarded as complementary rather than unique mediators of spatial communication. Their distinguishing feature is the membrane‐protected and coordinated delivery of selected proteins, lipids and nucleic acids to recipient cells, which may extend or modify the effects of soluble SASP signalling (Ziglari et al. 2025; Almeida and Melo 2025; Kalluri and McAndrews 2023).

### 6.3 Conceptual Framework: Placing senEVs Within the Cancer‐Immunity Cycle

The cancer‐immunity cycle is used here as a conceptual mapping tool rather than as an experimentally established senEV‐specific pathway. Most evidence for the individual steps derives from broader EV and tumour‐immunology studies (Ahn et al. 2025; Kuang et al. 2025; Spada and Ganguly 2025; Yue et al. 2023; Wolfers et al. 2001; Obregon et al. 2006; Van Niel et al. 2003; Segura et al. 2007; Nolte‐’t Hoen et al. 2009; Sheldon et al. 2010), whereas direct senEV evidence is currently strongest for antigen‐presenting‐cell recruitment (Ziglari et al. 2025) and PMN‐MDSC remodelling (Lai et al. 2025). To avoid reducing immune effects to a generalized ‘enhancement/suppression’ framework, this section adopts the process‐based framework of the cancer‐immunity cycle (antigen release → presentation → T‐cell priming → infiltration → killing) to locate the steps at which senEVs may intervene (Ahn et al. 2025; Kuang et al. 2025; Spada and Ganguly 2025; Yue et al. 2023; Wolfers et al. 2001; Obregon et al. 2006; Van Niel et al. 2003; Segura et al. 2007; Nolte‐’t Hoen et al. 2009; Sheldon et al. 2010): (a) **Antigen and danger‐signal release**: senEVs may carry tumour‐associated antigen fragments, stress proteins, or nucleic acid danger signals, thereby increasing the density of signals that can be read by the immune system (Wolfers et al. 2001); (b) **Antigen presentation and priming**: after uptake of senEVs by dendritic cells (DCs) or macrophages, their maturation, costimulatory‐molecule expression, and cross‐presentation efficiency may be altered (Obregon et al. 2006; Van Niel et al. 2003; Segura et al. 2007); (c) **Infiltration and effector execution**: senEVs may indirectly change immune‐cell migration through cargos related to chemotaxis/adhesion, and may also reduce effector‐cell function through suppressive molecules or metabolic stress signals (Nolte‐’t Hoen et al. 2009; Sheldon et al. 2010); (d) **Therapeutic response**: the role of EVs in resistance to immunotherapy has been systematically summarized, but whether senEVs contribute to this step remains a candidate relationship that requires direct testing (Kalluri and McAndrews 2023; Yue et al. 2023).

The value of this framework is that it allows us to decompose the phenomenon of ‘the same senEVs producing opposite outcomes’ into multiple testable node‐level questions—for example, whether in a given context senEVs mainly affect DC cross‐presentation, the myeloid suppressive network, or the threshold for effector T‐cell exhaustion. Differences at these nodes in turn determine whether intervention strategies should target biogenesis, cargo, or recipient cells (Choezom and Gross 2022; Hitomi et al. 2020; Menck et al. 2017; Pacifico et al. 2024).

### 6.4 Innate Immunity: Established senEV Effects and Proposed Responses of DCs, Macrophages, Neutrophils and PMN‐MDSCs

Evidence from broader EV studies suggests that EVs can influence dendritic‐cell maturation and antigen presentation (Wolfers et al. 2001; Obregon et al. 2006; Van Niel et al. 2003; Segura et al. 2007), whereas direct senEV evidence currently demonstrates recruitment of MHC‐II‐positive antigen‐presenting cells but does not yet establish all of the proposed effects on dendritic‐cell cross‐presentation (Ziglari et al. 2025). Innate immune cells are often the primary ‘readers’ of EVs and are also key amplifiers of immune‐outcome directionality. In this review, they are divided into four cell groups that merit parallel discussion: DCs, macrophages, neutrophils and PMN‐MDSCs.

1) Proposed dendritic‐cell effects: Maturation and cross‐presentation

Evidence that senEVs directly enhance or suppress dendritic‐cell maturation and cross‐presentation remains limited. Based on broader EV studies, senEV‐associated danger signals may promote DC maturation and subsequent T‐cell priming (Wolfers et al. 2001; Segura et al. 2007), whereas suppressive cargo could have the opposite effect; On the other hand, if senEVs carry immunosuppressive cargos or tolerance‐inducing metabolic/lipid signals, DCs may remain in a low‐maturation state, resulting in failed T‐cell priming. This is precisely the earliest branching point in the immunity cycle.

2) Macrophages: A shift from clearance/inflammation toward immunosuppressive phenotypes

Macrophages display marked functional plasticity in response to tissue‐derived signals. Miyazoe et al. (2020) showed that EVs released by senescent hepatic stellate cells increased epidermal growth factor (EGF) secretion from differentiated THP‐1 macrophage‐like cells and thereby enhanced hepatoma‐cell viability (Miyazoe et al. 2020). In a different context, Alibhai et al. (2020) found that aging‐ and senescence‐associated circulating EVs attenuated macrophage responses to lipopolysaccharide while increasing phagocytic activity (Alibhai et al. 2020). These findings indicate that senescence‐associated EVs can alter macrophage secretory and phagocytic functions, although whether the resulting response promotes tumour growth or senescent‐cell clearance depends on EV source, cargo and microenvironmental context.

3) Neutrophils and PMN‐MDSCs: mtDNA/nucleic acid signals may push innate immunity toward the suppressive end

As noted above, mtDNA signals released by senescent tumour cells can enhance PMN‐MDSC‐driven immunosuppression through a cGAS–STING‐related mechanism, thereby promoting immune escape (Lai et al. 2025). This suggests that when senEVs/related EV cargos are dominated by nucleic acid and mitochondrial stress information, recipient myeloid cells do not necessarily move toward a pro‐inflammatory antitumour state, but may instead shift toward suppressive tolerance, coupled to recurrence risk during the post‐treatment phase (Lai et al. 2025).

4) Conceptual implication for post‐treatment recurrence: Myeloid networks as potential amplifiers of immunosuppression

From a systems perspective, myeloid cells, especially MDSCs, can stabilize chronic immunosuppression. Once EV‐mediated signalling has reprogrammed these cells toward an immunosuppressive phenotype, senescent‐cell clearance may remain impaired even in the presence of effector T cells because antigen presentation is reduced, the metabolic environment is suppressive, and immune‐checkpoint pathways are enhanced. This provides an immune‐niche‐level basis for understanding poor responses/acquired resistance to immunotherapy.

### 6.5 Adaptive Immunity: The Connection Point Between T Cells and Natural Killer (NK) Cells in Senescence Surveillance—Enhancement or Suppression?

Adaptive immunity is the final execution layer that determines whether clearance truly occurs (Kang et al. 2011; Xue et al. 2007). Kang et al. (2011) demonstrated that CD4+ T cells mediated the clearance of premalignant senescent hepatocytes, thereby limiting liver cancer development (Kang et al. 2011). Xue et al. (2007) further showed that p53 restoration induced senescence and an innate immune response associated with the clearance of murine liver carcinomas (Xue et al. 2007).

Direct evidence that senEVs themselves broadly enhance or suppress T‐cell and NK‐cell functions remains limited. The following two modes should therefore be regarded as working hypotheses derived from senescence‐surveillance studies and the broader EV literature. On this basis, the effects of senEVs on T cells/NK cells may be understood in two opposite modes: (a) **Enhancement of transmissible immunity**: When senEVs carry stress‐related cues or cargos that promote antigen presentation, they may enhance T‐cell priming, improve infiltration efficiency, or support effector maintenance, thereby promoting clearance and suppressing recurrence (Wolfers et al. 2001; Obregon et al. 2006; Van Niel et al. 2003; Segura et al. 2007; Nolte‐’t Hoen et al. 2009); (b) **Promotion of immunosuppression and metabolic dysfunction**: When senEVs carry immunosuppressive cargo that inhibits T‐cell activation or enhances immune‐checkpoint signalling, or when they transmit metabolic and oxidative stress signals, they may reduce T‐cell proliferation, cytokine production, and cytotoxicity, promote T‐cell exhaustion, or decrease NK‐cell cytotoxicity, thereby facilitating immune escape and treatment resistance (Kalluri and McAndrews 2023; Ahn et al. 2025; Kuang et al. 2025; Spada and Ganguly 2025; Yue et al. 2023).

Accordingly, the net effect of senEVs on adaptive immunity should be understood more as the sum of multiple nodes (presentation, priming, infiltration, suppressive axes), rather than as the linear effect of a single molecule.

### 6.6 Potential Links Between senEVs, Immune Escape and Responses to Immunotherapy

Direct evidence that senEVs broadly mediate resistance to immune‐checkpoint therapy through PD‐L1 or mitochondrial cargo‐containing EVs remains limited. PD‐L1 is discussed here as contextual evidence from tumour‐derived EV studies and is not presented as a demonstrated senEV cargo. This subsection therefore outlines candidate mechanisms derived primarily from broader studies of tumour‐derived EVs and EVs containing mitochondrial components (Kalluri and McAndrews 2023; Ahn et al. 2025; Kuang et al. 2025; Spada and Ganguly 2025; Yue et al. 2023; Iorio et al. 2024; Sheng et al. 2025; Li et al. 2024; Wang et al. 2025). Among senEV‐specific studies, the strongest current evidence is that mtDNA‐containing EVs released by senescent tumour cells enhance PMN‐MDSC‐mediated immunosuppression (Lai et al. 2025).

Three candidate, potentially interacting pathways can be considered: (a) **Enhancement of checkpoint axes**: EVs may directly carry checkpoint‐related molecules or induce recipient cells to upregulate suppressive axes, thereby weakening effector T‐cell killing; (b) **Oxidative stress and metabolic resetting**: Post‐treatment tumour microenvironments are often characterized by fluctuations in ROS levels and nutrient redistribution. If EVs transmit mitochondria‐related cargo or metabolic reprogramming signals, they may increase stress tolerance in tumour cells. In recipient immune cells, the potential consequences include reduced antigen presentation, impaired effector T‐cell proliferation and cytokine production and decreased cytotoxic activity; however, these senEV‐specific effects require further direct experimental validation (Carles‐Fontana et al. 2022; Gade et al. 2025; Lai et al. 2025; Iorio et al. 2024; Sheng et al. 2025; Li et al. 2024; Wang et al. 2025); (c) **Stabilization of myeloid suppressive networks**: When EV signals chronically favour MDSCs/suppressive macrophage networks, immunotherapy may remain ineffective even if some checkpoints are relieved, because the upstream problems of presentation and niche organization have not been corrected.

Therefore, the relationship between senEVs and responses to immunotherapy is unlikely to be a simple matter of enhancing or reducing efficacy; rather, it depends on which nodes in the immunity cycle are primarily targeted by senEVs: targeting presentation/priming may amplify efficacy, whereas targeting suppressive axes/myeloid networks may promote resistance.

### 6.7 Summary: Established Evidence, Working Hypotheses and Research Priorities

Taken together, experimentally supported senEV activities include the recruitment of MHC‐II‐positive antigen‐presenting cells and immune‐mediated clearance of senescent cells (Ziglari et al. 2025), modulation of macrophage functions in selected senescence models (Alibhai et al. 2020; Miyazoe et al. 2020), IFITM3‐dependent propagation of paracrine senescence (Borghesan et al. 2019), GPX4‐dependent ferroptosis resistance and chemoresistance (Jia et al. 2026) and mtDNA‐dependent activation of PMN‐MDSC immunosuppression (Lai et al. 2025). In contrast, the proposed acute‐to‐chronic temporal transition, the cancer‐immunity‐cycle framework, broad regulation of T‐cell and NK‐cell functions, and mitochondrial stress as a general determinant of senEV immune directionality remain working hypotheses. Future studies should directly compare senEV cargo composition, recipient‐cell targeting, and immune outcomes across different senescence inducers, donor‐cell types, therapeutic contexts and sampling times. These evidence gaps provide the basis for the translational strategies and future research priorities discussed in Sections 7 and 8.

## 7 Candidate Translational Directions and Current Limitations

### 7.1 Candidate Intervention Levels: EV Biogenesis, Pathogenic Cargo and Senescent Source Cells

Based on current preclinical evidence, three candidate intervention levels can be considered. First, inhibition of senEV biogenesis or release may be explored by targeting pathways such as the DDR–ceramide–nSMase2 axis (Choezom and Gross 2022; Hitomi et al. 2020; Menck et al. 2017; Pacifico et al. 2024). Second, cargo‐specific interventions may be tested in models in which the effects of EphA2, SERPINE1, IFITM3, GPX4, or mtDNA have been experimentally demonstrated (Lai et al. 2025; Zhang et al. 2024; Takasugi et al. 2017; Borghesan et al. 2019; Jia et al. 2026). Third, eliminating senescent source cells may reduce the production of pathogenic senEVs, although the relative contribution of EV‐dependent and soluble SASP‐dependent effects would need to be distinguished (Dong et al. 2024; Song et al. 2023; Ding et al. 2025; Wang et al. 2024; Liu et al. 2024; Lai et al. 2025; Zhang et al. 2024; Saleh 2024; Xiao et al. 2023). These approaches remain preclinical, and their selectivity, safety, and generalizability have not been established.

These three intervention levels are not necessarily mutually exclusive. However, the temporal evolution of senEV composition and function after treatment has not been systematically defined, and a general transition from early immune‐stimulatory senEVs to chronic immunosuppressive senEVs has not been experimentally established. Consequently, current evidence does not support recommending a specific therapeutic window for EV‐pathway inhibition. Longitudinal studies are required before timing‐based intervention strategies can be proposed.

### 7.2 Feasibility and Risks of Intervening in EV Pathways

At the level of experimental tractability, nSMase2 represents a candidate node for mechanistic and preclinical investigation because it is associated with ceramide‐dependent EV biogenesis and can be pharmacologically or genetically modulated (Choezom and Gross 2022; Hitomi et al. 2020; Menck et al. 2017; Trajkovic et al. 2008). Hitomi et al. (2020) showed that DNA damage increased senescence‐associated EV release through a ceramide‐dependent mechanism involving reduced SMS2 expression and increased neutral sphingomyelinase 2 (nSMase2) expression (Hitomi et al. 2020). The same study indicated that this EV‐release pathway facilitated the removal of cytoplasmic DNA fragments and limited excessive inflammatory responses (Hitomi et al. 2020).

However, systemic inhibition of EV release carries potential risks because EV‐mediated communication contributes not only to tumour biology but also to normal tissue homeostasis and immune regulation. In senescent cells, EV release may also facilitate cytoplasmic DNA removal and limit excessive intracellular inflammatory signalling (Hitomi et al. 2020). Therefore, global and sustained inhibition of EV release would require particular caution. Future preclinical studies should address three issues: (a) temporal characterization—senEV abundance, cargo composition, and biological activity should be measured longitudinally before an intervention time point is selected; (b) delivery specificity—local or tumour‐enriched delivery should be evaluated to reduce interference with physiological EV functions (Choezom and Gross 2022; Hitomi et al. 2020; Menck et al. 2017; Pacifico et al. 2024); and (c) pharmacodynamic and safety assessment—senEV abundance, EV‐associated mtDNA, recipient‐cell responses, and markers of myeloid immunosuppression should be monitored to determine target engagement and potential adverse effects (Lai et al. 2025; Welsh et al. 2024; Thery et al. 2018). These studies should establish whether selective EV‐pathway inhibition improves therapeutic outcomes without disrupting normal tissue homeostasis or beneficial immune clearance.

### 7.3 Candidate Biomarkers and Validation Requirements for senEV Liquid Biopsy

Here, ‘senescent‐cell burden’ and ‘immunosuppressive risk’ are used as exploratory concepts rather than validated clinical variables. The former refers to the abundance of senescent tumour and stromal cells, whereas the latter refers to the possibility that their EV output contributes to myeloid immunosuppression or immune escape.

Although senEV‐associated liquid biopsy is potentially informative, its clinical utility has not yet been established. Current studies primarily identify candidate analytical dimensions that require further validation. For senEV‐based liquid biopsy, reliance on a single biomarker is unlikely to capture the heterogeneity of senescent‐cell‐derived EVs; a more informative approach is to develop an integrated biomarker panel (Meng et al. 2023; Lee et al. 2022). The more representative dimensions at present include:

1. The surfaceome dimension: Infer tissue distribution and target‐cell spectra based on differences in the surfaceome of senescent EVs. Meng et al. (2023) showed that senescent‐cell‐derived EVs had a surface protein profile distinct from that of proliferating‐cell‐derived EVs. They further identified dipeptidyl peptidase‐4 (DPP4) as a negative regulator of EV uptake: DPP4 on the surface of senescent‐cell‐derived EVs reduces their internalization by proliferating recipient cells (Meng et al. 2023);
2. The physical‐properties dimension: Use ‘label‐free fingerprints’ such as nanomechanical properties, electrical properties and spectroscopy as supplementary dimensions, thereby improving the resolution for discriminating subtype differences. Lee et al. (2022) reported that senescent‐cell‐derived sEVs had slightly stiffer surfaces but softer cores than non‐senescent‐cell‐derived sEVs and also exhibited differences in surface potential and Raman spectral features (Lee et al. 2022);
3. The mitochondrial cargo‐containing EV dimension: EV‐associated mitochondrial DNA (mtDNA) and, where feasible, other mitochondrial components should be quantified as candidate indicators of immunosuppressive remodelling. Lai et al. (2025) showed that senescent tumour cells package mtDNA into EVs, which are selectively transferred to PMN‐MDSCs. Following uptake, EV‐associated mtDNA activates cGAS–STING–NF‐κB signalling and enhances the immunosuppressive activity of PMN‐MDSCs, thereby promoting tumour progression (Lai et al. 2025). These findings support EV‐associated mtDNA as a candidate biomarker of senEV‐related immune risk. Nevertheless, clinical assays should distinguish EV‐associated mtDNA from freely circulating mtDNA and mtDNA released through nonspecific cell damage, and longitudinal studies are needed to determine whether changes in these fractions predict treatment resistance or recurrence;
4. Methodological standardization according to the minimal information for studies of extracellular vesicles (MISEV) guidelines: The entire workflow should follow the updated MISEV2023 recommendations and standardized pre‐analytical reporting requirements (Welsh et al. 2024; Thery et al. 2018). MISEV2023 provides recommendations on EV nomenclature, sample collection and preprocessing, separation, characterization, and functional validation, and defines EVs as naturally released particles delimited by a lipid bilayer that cannot replicate (Welsh et al. 2024). Adherence to these recommendations is essential to prevent cell debris, non‐vesicular particles, and lipoproteins from being misidentified as EV signals, thereby reducing false‐positive findings and improving reproducibility.

These analytical dimensions should not yet be combined into a clinical ‘senescence burden + immune risk’ score. Before such a score can be proposed, candidate markers must be validated in independent patient cohorts, linked to experimentally confirmed senescent cellular sources, distinguished from EVs released by non‐senescent cells and from non‐vesicular contaminants, and prospectively associated with treatment response or recurrence. It will also be necessary to determine whether a multimarker senEV panel provides additional predictive value beyond established clinical and molecular biomarkers.

## 8 Conclusion, Outstanding Questions and Research Priorities

Cancer therapy can induce senescence in surviving tumour and stromal cells and thereby alter both soluble and EV‐associated secretory signalling (Dong et al. 2024; Song et al. 2023; Ding et al. 2025; Wang et al. 2024; Tanaka and Takahashi 2021; Liu et al. 2024). Direct experimental studies demonstrate that senEVs can promote immune‐mediated clearance of senescent cells (Ziglari et al. 2025), enhance recipient tumour‐cell proliferation through EphA2 (Takasugi et al. 2017), promote colorectal cancer progression through EV‐associated SERPINE1 (Zhang et al. 2024), propagate paracrine senescence partly through IFITM3 (Borghesan et al. 2019), confer ferroptosis resistance and chemoresistance through GPX4 transfer from senescent endothelial cells (Jia et al. 2026), and enhance PMN‐MDSC‐mediated immunosuppression through EV‐associated mtDNA (Lai et al. 2025). These findings establish functional heterogeneity among senEVs. However, many proposed connections among senEV biogenesis, mitochondrial quality control, immune remodelling, therapeutic resistance, and recurrence remain incompletely validated and rely partly on evidence from broader EV or senescence research. Mitochondrial stress should therefore be regarded as an emerging, testable framework rather than an established general regulator of senEV function.

The apparently contradictory effects of senEVs likely reflect differences in senescence inducer, donor‐cell type, cargo composition, recipient‐cell population and immune context. Time after treatment may also contribute, but a general transition from acute immune‐stimulatory senEVs to chronic immunosuppressive senEVs has not yet been demonstrated. Future studies should therefore focus on functionally and experimentally defined senEV populations rather than assuming that total EV abundance or treatment time alone predicts biological outcome.

Looking ahead, there are at least five key questions in this field that most urgently need to be answered and are also the most likely to directly drive translation:

1) Can senEV subtypes be matched one‐to‐one with specific functions?

The opposing biological effects reported for senEVs can be partly attributed to heterogeneity among EV subpopulations (Ziglari et al. 2025; Almeida and Melo 2025). The next step is to move from describing the overall effect of EVs to a refined mapping of subtype–cargo–recipient cell–outcome: which surfaceome or physical fingerprints correspond to clearance‐promoting effects, and which correspond to suppression‐promoting effects? Which are the true senEV subpopulations carrying high‐risk cargo profiles that should be prioritized for interception (Meng et al. 2023; Lee et al. 2022)? Existing studies on the DPP4 surfaceome and nanophysical properties have already shown that differences among senEVs can be detected, but they are still far from establishing a one‐to‐one correspondence between subtype and function.

2) What is the route of origin of mitochondrial cargo: Is secretory mitophagy the main pathway or only a special case?

Mitochondrial cargo may enter EVs through multiple biogenetic routes, including secretory mitophagy, rerouting through the endosomal system and budding from membrane microdomains (Carles‐Fontana et al. 2022; Gade et al. 2025; Sheng et al. 2025). Clarifying which pathway predominates under specific treatment conditions and in specific cell types may inform whether intervention should target EV biogenesis or cargo loading (Welsh et al. 2024; Thery et al. 2018; Sheng et al. 2025). In particular, more rigorous operational definitions and validation chains are needed to distinguish genuine loading from isolation‐related contamination (Ahn et al. 2025; Dou et al. 2017; Gluck et al. 2017). Current studies on secretory mitophagy and mitochondria‐encapsulating EVs provide a mechanistic entry point to this question, but a unified model is still lacking.

3) Which immune‐cell populations respond to senEVs, and in which anatomical compartments do these interactions occur?

At present, many conclusions remain at the broad level of immune activation or suppression. Future studies should use spatially resolved approaches to determine where senEV–immune‐cell interactions occur—for example, within the tumour, tumour‐draining lymph nodes, or peripheral tissues—and how these interactions vary across anatomical locations. These studies should also define the response of each immune‐cell population to senEV cargo, including antigen uptake and presentation by dendritic cells, pro‐ or anti‐inflammatory polarization of macrophages, immunosuppressive activation of PMN‐MDSCs, and activation or inhibition of T‐cell responses (Wolfers et al. 2001; Obregon et al. 2006; Van Niel et al. 2003; Segura et al. 2007; Nolte‐’t Hoen et al. 2009; Sheldon et al. 2010). The answers in terms of space and time window will directly determine the route and timing design of combination therapy (Ziglari et al. 2025; Almeida and Melo 2025). At present, the most direct mechanistic evidence for senEV‐mediated immunosuppression comes from Lai et al. (2025), who showed that mtDNA packaged into EVs released by senescent tumour cells is transferred to PMN‐MDSCs and enhances their immunosuppressive activity through cGAS–STING–NF‐κB signalling (Lai et al. 2025). However, a more complete cellular atlas and spatial map of senEV recipient cells remain to be established.

4) How can senEVs be distinguished from EVs Released by non‐senescent tumour or stromal cells and from non‐vesicular particles in clinical samples?

Clinical specimens contain a heterogeneous mixture of EVs released by senescent and non‐senescent tumour and stromal cells, together with cell debris, lipoproteins and inflammation‐associated non‐vesicular particles. Therefore, classification of an EV population as senescence‐associated should require evidence linking it to a senescent cellular source, rather than tumour origin alone. Standardized sample preprocessing, EV separation, characterization, and functional validation according to MISEV2023 are needed to reduce methodological artifacts and improve comparability (Welsh et al. 2024; Thery et al. 2018). Biomarker development should then evaluate whether integrated profiles combining surface proteins, biophysical properties, and mitochondrial cargo can distinguish senEV‐enriched populations and estimate senescent‐cell burden and immunosuppressive risk (Lai et al. 2025; Meng et al. 2023; Lee et al. 2022).

5) How do senEV composition and function change over time after therapy, and are these temporal patterns reproducible across models?

Longitudinal studies should characterize senEV abundance, subtype distribution, cargo composition, cellular origin, recipient‐cell targeting, and biological activity at multiple time points after treatment. These studies should compare different therapies, tumour types, senescence inducers, and immune contexts to determine whether any reproducible temporal pattern exists. Only after such patterns have been experimentally demonstrated should studies evaluate whether the timing of nSMase2 inhibition, cargo‐specific blockade, or senescent‐cell elimination alters therapeutic outcomes (Lai et al. 2025; Zhang et al. 2024; Takasugi et al. 2017; Choezom and Gross 2022; Hitomi et al. 2020; Menck et al. 2017; Pacifico et al. 2024).

In summary, the immediate priority for the senEV field is not clinical implementation but rigorous causal and methodological validation. This includes establishing reproducible definitions of senEV populations, distinguishing senEV‐specific mechanisms from general EV biology, identifying causal cargo–recipient‐cell relationships, performing longitudinal and cross‐model comparisons, and developing assays that can reliably attribute circulating EV signals to senescent cellular sources. Only after these requirements are met will it be possible to determine whether senEV‐associated cargos can serve as clinically useful biomarkers or whether selective manipulation of senEV pathways can improve cancer‐treatment outcomes without disrupting physiological EV communication or beneficial senescence surveillance.

## Author Contributions

**Yongrui Qian**: conceptualization, methodology, writing – review and editing, writing – original draft, software, data curation. **Zirui Weng**: visualization, investigation, validation. **Na Li**: validation, investigation. **Yun Zhou**: funding acquisition, project administration, supervision. **Hailong Pei**: project administration, funding acquisition, supervision.

## Ethics Statement

The authors have nothing to report.

## Consent

The authors have nothing to report.

## Conflicts of Interest

The authors declare no conflicts of interest.

## Data Availability

The data that support the findings of this study are available from the corresponding author upon reasonable request.
